# Partial Fourier in the presence of respiratory motion in prostate diffusion-weighted echo planar imaging

**DOI:** 10.1007/s10334-024-01162-x

**Published:** 2024-05-14

**Authors:** Sean McTavish, Anh T. Van, Johannes M. Peeters, Kilian Weiss, Felix N. Harder, Marcus R. Makowski, Rickmer F. Braren, Dimitrios C. Karampinos

**Affiliations:** 1 Department of Diagnostic and Interventional Radiology, School of Medicine, Technical University of Munich, Ismaninger Str. 22, 81675 Munich, Germany; 2 Philips Healthcare, Best, Netherlands; 3 Philips GmbH, Hamburg, Germany

**Keywords:** Diffusion-weighted imaging, Prostate imaging, Apparent diffusion coefficient (ADC) mapping, Single-shot echo planar imaging (EPI), Multi-shot EPI

## Abstract

**Purpose:**

To investigate the effect of respiratory motion in terms of signal loss in prostate diffusion-weighted imaging (DWI), and to evaluate the usage of partial Fourier in a free-breathing protocol in a clinically relevant *b*-value range using both single-shot and multi-shot acquisitions.

**Methods:**

A controlled breathing DWI acquisition was first employed at 3 T to measure signal loss from deep breathing patterns. Single-shot and multi-shot (2-shot) acquisitions without partial Fourier (no pF) and with partial Fourier (pF) factors of 0.75 and 0.65 were employed in a free-breathing protocol. The apparent SNR and ADC values were evaluated in 10 healthy subjects to measure if low pF factors caused low apparent SNR or overestimated ADC.

**Results:**

Controlled breathing experiments showed a difference in signal coefficient of variation between shallow and deep breathing. In free-breathing single-shot acquisitions, the pF 0.65 scan showed a significantly (*p* < 0.05) higher apparent SNR than pF 0.75 and no pF in the peripheral zone (PZ) of the prostate. In the multi-shot acquisitions in the PZ, pF 0.75 had a significantly higher apparent SNR than 0.65 pF and no pF. The single-shot pF 0.65 scan had a significantly lower ADC than single-shot no pF.

**Conclusion:**

Deep breathing patterns can cause intravoxel dephasing in prostate DWI. For single-shot acquisitions at a *b*-value of 800 s/mm^2^, any potential risks of motion-related artefacts at low pF factors (pF 0.65) were outweighed by the increase in signal from a lower TE, as shown by the increase in apparent SNR. In multi-shot acquisitions however, the minimum pF factor should be larger, as shown by the lower apparent SNR at low pF factors.

**Supplementary Information:**

The online version contains supplementary material available at 10.1007/s10334-024-01162-x.

## Introduction

Diffusion weighted imaging (DWI) is an important tool for the detection and characterization of lesions in the prostate [1, 2]. The apparent diffusion coefficient (ADC) value, which is derived from acquiring diffusion weighted images at different *b*-values, has also been shown to correlate with tumour aggressiveness [3–8]. The current prostate imaging reporting and data system (PI-RADS) guidelines recommend using a free breathing spin echo sequence with an echo planar imaging (EPI) readout for diffusion weighted imaging [9]. As prostate tumours can be small, there is an ongoing need for high resolution protocols [10]. However, increasing the resolution increases the length of the EPI readout, which in turn increases the echo time (TE) of the sequence and decreases the phase encoding bandwidth per pixel. Acquisitions with longer echo times have reduced SNR due to *T*_2_ decay [11]. Acquisitions with a lower phase encoding bandwidth suffer from stronger off-resonance effects.

Partial Fourier (pF) is one method that can be utilized to decrease the length of the EPI echo train, and, therefore, shorten the TE, whilst maintaining a high spatial resolution [12, 13]. Partial Fourier exploits the Hermitian symmetry of *k*-space by acquiring all *k*-space lines in one half of *k*-space, and a reduced number of *k*-space lines in the other half [14]. However, motion that occurs during the diffusion encoding gradients can cause the *k*-space centre to shift away from the centre of the EPI readout [15, 16]. If the motion is large enough, then the *k*-space centre can shift completely out of the sampled region of *k*-space, causing severe signal loss in the reconstructed images. Motion can also cause a reduction in the effective resolution of the image if the *k*-space shift causes high *k*-space frequencies to not be acquired. Since pF reduces the acquired number of *k*-space lines, it is, therefore, more sensitive to motion induced phase error effects and must be used with care. Storey et al. [15] showed severe artefacts caused by rigid body motion in the brain which were reduced with an adaptive homodyne algorithm. Chang et al. [17] extended the use of an adaptive homodyne algorithm to multi-shot acquisitions in the brain. Zhang et al. [18] and Geng et al. [19] were able to correct for severe motion artefacts with motion compensated waveforms whilst using a pF factor of 0.75 in the liver and pancreas, respectively. Sources of motion artefacts in the liver and pancreas can be from cardiac pulsation and respiration. Van et al. [16] showed that phase correction can reduce motion induced worm like artefacts and signal loss in a homodyne reconstruction in the liver. Filli et al. [20] used a pF factor of 5/8 in the breast but did not report any considerable motion artefacts.

Velocity compensated diffusion encoding waveform designs have been employed in other organs such as the liver [18, 21–28], pancreas [19], kidney [29] and heart [22, 30–33]. However, velocity compensated waveforms suffer from long echo times and the prostate is not typically considered to be associated with large amounts of motion [34, 35]. Previous work from our group has established a link between respiratory motion and phase errors in prostate DWI [35]. Asymmetric designs for the standard Stejskal–Tanner pulsed gradient spin echo (pgse) [36] waveform, such as the convex optimized diffusion encoding (CODE) waveform [22], have been employed in the prostate with eddy current-nulling (ENCODE) [37, 38], and remain another option for reducing the echo time in a DW-EPI sequence. However, asymmetric waveform designs can suffer from concomitant field effects [39–41], which can confound the calculation of the ADC value if they are not properly compensated for.

High-resolution EPI readouts suffer from geometric distortions in regions of differing magnetic susceptibility. As the prostate is in close vicinity to the rectum, large geometric distortions can be observed near the tissue-air boundary [42]. Multi-shot sequences have been proposed to reduce the impact of geometric distortions as a result of their increased phase encoding bandwidth [43–46]. Multi-shot sequences split the acquisition of *k*-space lines into multiple different excitations and require a method of navigation to correct for shot-to-shot phase inconsistencies caused by motion. Navigation methods include approaches acquiring a low-resolution navigator after the EPI readout of the imaging echo [47–53] and self-navigated approaches [54–60].

Whereas pF has previously been used in the prostate [38, 45, 61–65], there are no PI-RADS recommendations or consensus on the usage of pF [9, 66–68] and, to the best of our knowledge, there are no studies that have explicitly investigated how low of a pF factor one can use in both single-shot and multi-shot acquisitions in prostate DWI in a clinically relevant *b*-value range. The purpose of this work is to investigate if cases of deep respiratory motion can cause severe motion artefacts, and to evaluate if free-breathing protocols are significantly affected by respiratory motion, which can affect how small of a partial Fourier factor one can use in a clinically relevant *b*-value range using both single-shot and multi-shot acquisitions. The authors hypothesize that only deep respiratory motion is likely to cause large signal loss and, therefore, small pF factors can be used in a free-breathing clinically relevant protocol with normal breathing patterns.

## Methods

### In vivo MRI measurements

MRI measurements were performed in 10 healthy male volunteers (mean age, 30 ± 7 years) on a 3 T Ingenia Elition X scanner (Philips Healthcare, Best, Netherlands), with a maximum gradient amplitude of 45 mT/m and a maximum slew rate of 220 T/m/s. The built in 12-channel posterior and 16-channel anterior coil were used for signal reception. The study was approved by the local ethics commission and all volunteers consented for their participation in the study.

The multi-shot DWI acquisitions were based on the acquisition of a navigator echo for each shot, which was refocused from the imaging echo. The phase of the navigator was then used to correct for the phase variation between each shot during the reconstruction process in image space. Further details are given in [47]. All pF scans, both in-vivo and simulated, were reconstructed with a homodyne reconstruction [14]. Homodyne reconstruction restores only magnitude information, and the accompanied presented phase is the low resolution phase from the symmetrically sampled region [14].

All scans used a phase encoding direction of anterior–posterior (AP), a parallel imaging SENSE factor of *R* = 2 in the phase encoding (AP) direction, a voxel size of 2.0 × 2.0 × 3.5 mm^3^ and a FOV of 160 × 160 mm^2^.

### Controlled-breathing dynamic DWI measurements and analysis

To investigate how the magnitude of breathing affects the signal intensity in prostate DWI, an axial multi-slice controlled breathing pgse dynamic DWI scan was performed in an arbitrarily selected subset of 5 out of the 10 healthy volunteers for both single-shot and multi-shot (2-shot) acquisitions. The scan parameters are shown in Table 1. The TR = 3000 ms was chosen so as to avoid long scan times, allowing the subjects to keep a more consistent controlled breathing pattern. While the TR is shorter than the TR in the free-breathing partial Fourier DWI measurements, which will be described in the next section, the TR is not expected to play a significant role in the signal loss pattern caused by motion during the diffusion encoding period. The volunteer was instructed to breathe deeply and shallowly in separate scans.

**Table 1:** Scan parameters for the in vivo controlled-breathing dynamic DWI and free-breathing partial Fourier DWI measurements

Scan	pF factor	Number of shots	*b*-values (repetitions)	Diffusion encoding directions (right-left, anterior–posterior, superior-inferior)	TE/TR in ms (ms)	Number of slices	Bandwidth in PE direction	Matrix size	Number of phase encoding lines	Total scan time	Gradient first/second moment (s/mm/s^2^/mm)
*Controlled-breathing dynamic DWI*											
1	No pF	1	800 s/mm^2^ (40)	(0, 1, 0)	89/3000	4	20.9 Hz/pixel	80 × 79	69	2 min 6 s	1.01/0.059
2	No pF	2	800 s/mm^2^ (20)	(0, 1, 0)	70/3000	4	37.7 Hz/pixel	80 × 80	70	2 min 6 s	0.91/0.047
*Free-breathing partial Fourier DWI*											
1	No pF	1	100 (3), 800 (7) s/mm^2^	((− 0.5, − 1, − 1), (1, 0.5, − 1), (1, − 1, 0.5))	83/4500	26	21.8 Hz/pixel	80 × 79	69	2 min 29 s	0.97/0.052
2	0.75	1	100 (3), 800 (7) s/mm^2^	((− 0.5, − 1, − 1), (1, 0.5, − 1), (1, − 1, 0.5))	62/4500	26	22.4 Hz/pixel	80 × 79	52	2 min 29 s	0.89/0.039
3	0.65	1	100 (3), 800 (7) s/mm^2^	((− 0.5, − 1, − 1), (1, 0.5, − 1), (1, − 1, 0.5))	53/4500	26	21.5 Hz/pixel	80 × 79	45	2 min 29 s	0.81/0.032
4	No pF	2	100 (3), 800 (7) s/mm^2^	((− 0.5, − 1, − 1), (1, 0.5, − 1), (1, − 1, 0.5))	63/4500	26	35.9 Hz/pixel	80 × 80	70	4 min 57 s	0.85/0.036
5	0.75	2	100 (3), 800 (7) s/mm^2^	((− 0.5, − 1, − 1), (1, 0.5, − 1), (1, − 1, 0.5))	51/4500	26	37.3 Hz/pixel	80 × 80	52	4 min 57 s	0.79/0.030
6	0.65	2	100 (3), 800 (7) s/mm^2^	((− 0.5, − 1, − 1), (1, 0.5, − 1), (1, − 1, 0.5))	48/4500	26	39.0 Hz/pixel	80 × 80	46	4 min 57 s	0.77/0.029

An ROI was drawn over the whole prostate for each slice and the same ROI was used for each repetition and scan. The data were acquired without pF, and the pF factors of (0.75 0.70 0.65 0.60 0.55) were simulated by removing the corresponding number of *k*-space lines for the desired pF factor, however, the effect of a reduced TE was not simulated. The purpose of the simulated pF was to investigate if removing *k*-space lines caused the *k*-space centre to no longer be within the sampled *k*-space range. The coefficient of variation (CV) of the signal was calculated by finding the standard deviation of the ROI mean signal magnitude over all repetitions, then dividing by the mean of the ROI mean signal magnitude over all repetitions. As motion can cause signal loss artefacts, a higher signal CV implies a higher incidence of motion induced signal loss. The CV over all subjects was calculated from [69]

$${\text{CV}}_{{\text{subjects}}} = \frac{{\sqrt {{\sum_{j = 1}^m {\frac{{{\text{Signal}}_{SD_j } }^2}{m}} }} }}{{\sum_{j = 1}^m {\frac{{{\text{Signal}}_{{\text{mean}}_j } }}{m}} }},$$

where ${\text{CV}}_{{\text{subjects}}}$ is the signal coefficient of variation over all subjects, ${\text{Signal}}_{{\text{SD}}}$ is the standard deviation of the signal for a single subject, ${\text{Signal}}_{{\text{mean}}}$ is the mean of the signal for a single subject, and m is the number of subjects.

### Free-breathing partial Fourier DWI measurements and analysis

To investigate the effect of pF on single-shot and multi-shot prostate DWI measurements, axial multi-slice free-breathing DWI scans were performed in all 10 healthy subjects with *b*-values of (100, 800) s/mm^2^ as recommended by the PI-RADS guidelines [9]. The scan parameters are shown in Table 1. Diffusion directions (− 0.5, − 1, − 1), (1, 0.5, − 1) and (1, − 1, 0.5) will from now on be referred to as dir 1, dir 2 and dir 3, respectively. The acquisition order was as follows (from inner to outer loop): slices, repetitions, shots, diffusion directions, *b*-values. However, it is motion during the diffusion encoding (which is of the order of 50 ms) that causes intravoxel dephasing and, therefore, signal loss.

Apparent SNR maps of the *b* = 800 s/mm^2^ images were calculated by first averaging each of the 7 repetitions per diffusion direction along the diffusion direction dimension, to give a total of 7 averaged images. The final SNR map was calculated by then taking a pixel by pixel mean divided by the standard deviation of the signal magnitude over all 7 averaged images. For the apparent SNR maps per diffusion direction, the final SNR map was calculated by taking a pixel by pixel mean divided by the standard deviation of the signal magnitude over all 7 repetitions per diffusion direction. The apparent SNR maps were in effect a measure of signal variability. Although a shorter TE from using pF causes the overall signal to increase, the *b* = 800 s/mm^2^ images are sensitive to motion as a result of the diffusion encoding gradients. Any motion that causes signal loss would, therefore, reduce the apparent SNR, potentially causing pF acquisitions which have a shorter TE to have lower apparent SNR if the pF sequence parameters caused an increased sensitivity to motion induced signal loss. A similar calculation of the apparent SNR was performed in [29]. The ADC maps were calculated from a monoexponential fit. Signal loss due to motion causes an overestimation of the ADC value. Acquisitions which are sensitive to motion would, therefore, have a higher ADC than those which are less sensitive to motion if the motion is severe enough and happens frequently enough. ROIs were drawn in 3 adjacent slices in the mid prostate, with 2 ROIs drawn per slice in the left and right sides of the whole peripheral zone (PZ) of the prostate and 1 ROI drawn per slice in the transitional zone (TZ) of the prostate. A standard anatomical *T*_2_-weighted image was used for guidance in the ROI drawing. All ROIs were drawn by a doctoral student and checked by a radiologist with 6 + years of experience. For each scan, the ROIs were then adjusted depending on the position of the prostate. The ROIs were applied to the apparent SNR and ADC maps, and the mean value over all slices and ROIs were calculated. Simulated pF data were generated from the scans which were acquired without pF encoding, as described in the previous section describing the controlled-breathing dynamic full-Fourier DWI measurement and analysis. However, in the free-breathing partial Fourier DWI measurement simulations, the SNR maps were adjusted for reduction in TE using TEs from the scanner and *T*_2_ values of 70.6 ms and 57.4 ms for the PZ and Central Gland (CG), respectively, as given in [70]. For simulated pF factors of 0.75, 0.70, 0.65 and 0.60, the single-shot TEs were 62, 57, 53 and 49 ms, respectively; the multi-shot TEs were 51, 49, 48 and 46 ms, respectively. The apparent SNR and ADC values over all subjects were first tested for normal distribution with a Kolmogorov–Smirnov test, then tested for statistical significance with a paired t-test. Statistical significance was defined as *p* < 0.05.

### Calculation of motion sensitivity

While Table 1 also shows the first and second gradient moments, which give a measure of the motion sensitivity of the sequence, in the case of pF acquisitions the gradient moments may not describe the full picture, as the acquired region of *k*-space is asymmetric, meaning that the impact of a *k*-space shift depends on the direction of the shift. From Storey et al. [15], the minimum angular velocity of the prostate at which signal loss can occur is given by

1 $$\left| {\Omega_z } \right| > \frac{k_0 }{{\gamma |G|\delta_{{\text{dur}}} \Delta_{{\text{sep}}} }}$$

where $\left| {\Omega_z } \right|$ is the angular velocity, $k_0$ is the *k*-space line representing the symmetric part of *k*-space in a pF acquisition, $\gamma$ is the gyromagnetic ratio, $\left| G \right|$ is the gradient strength, $\delta_{{\text{dur}}}$ is the duration of the diffusion gradient lobe and $\Delta_{{\text{sep}}}$ is the temporal separation of the leading edges diffusion gradient lobes. The minimum angular velocity at which signal loss can occur was calculated from the sequence parameters to give a measure of the motion sensitivity of each sequence.

## Results

### Controlled-breathing dynamic DWI results

**Fig. 1:**
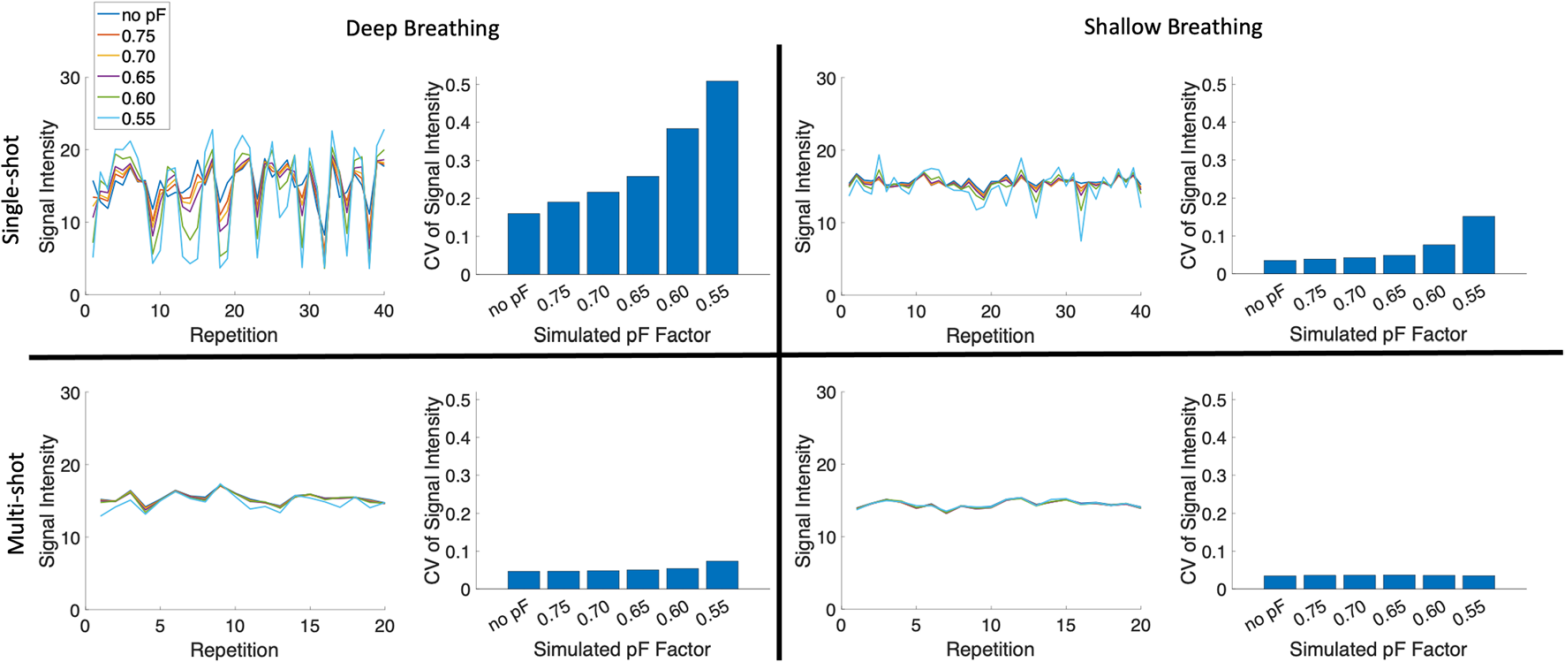
Signal intensity and CV of signal intensity over an ROI over all repetitions for a range of simulated partial Fourier factors in a single volunteer. Both deep and shallow breathing acquisitions were performed, for both single-shot and multi-shot (2 shots). The multi-shot acquisition was 2 shots for 20 repetitions and, therefore, had the same number of total acquisitions as the 40 repetition single-shot acquisition. In the single-shot deep breathing case, there is a clear increase in signal variation as the partial Fourier factor is decreased, whereas in the single-shot shallow breathing case, the signal variations are much smaller compared to the deep breathing case. There are also some data points where using a low pF causes a large signal drop compared to no pF, which may be due to motion. The signal variation in the deep breathing multi-shot case is smaller than the single-shot deep breathing case, which may be due to the fact each repetition is the combination of 2 separate acquisitions, which has an averaging effect. There is not much signal variation in the multi-shot shallow breathing case

**Fig. 2:**
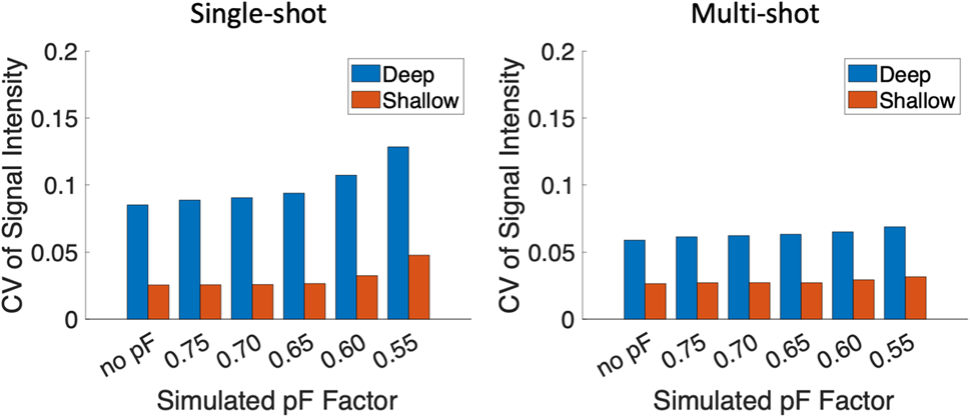
CV of signal intensity over all subjects, with the deep breathing results shown in blue and shallow breathing results shown in red. In both single-shot and multi-shot cases, deep breathing has a higher CV than shallow breathing. The simulated pF factor of 0.55 also has the highest signal CV in all cases. The multi-shot cases in general have lower signal CVs than the equivalent single-shot cases, probably due to the averaging effect of acquiring multiple shots

Controlled-breathing dynamic DWI scans were performed to determine if breathing motion can cause intravoxel dephasing. Supplementary Material Fig. S1 shows example images from a single subject, and Fig. 1 shows the single-shot and multi-shot controlled breathing (deep vs. shallow) results for one subject. In the single-shot case, the DWI signal CV without pF was larger with deep breathing than with shallow breathing, most likely because of increased signal loss due to motion during the diffusion encoding of individual measurements, which lead to a larger signal variation over all measurements. As the pF factor was reduced, the signal CV increased. In the deep-breathing single-shot case, there were much larger differences in signal CV between subsequent pF factors as the pF factor was reduced below 0.65, and there were many data points where there was a large drop in signal when using low pF factors in comparison to no pF, suggesting that breathing motion can cause intravoxel dephasing which can be exacerbated using lower pF factors in prostate DWI. Supplementary Material Fig. S1 shows many cases of severe signal loss in the single-shot deep breathing case. In the multi-shot case, the signal CV was low for both deep and shallow breathing in comparison to the single-shot case. However, as the reconstructed images are from 2 shots, there is an averaging effect in the signal intensity from combining the shots, which will then have an averaging effect in the signal CV. Figure 2 shows the signal CV over all subjects. In general, the signal CV is higher for deep breathing in comparison to shallow breathing, in both single-shot and multi-shot cases. Lower pF values also in general have higher signal CV values than higher pF values.

The minimum prostate angular velocities at which signal loss could occur, calculated from Eq. 1 were 14.2°/s and 15.7°/s for the single-shot and multi-shot scans, respectively.

### Free-breathing DWI partial Fourier scan results

In the single-shot scans, the minimum prostate angular velocities at which signal loss could occur, calculated from Eq. 1, were 14.8°/s, 8.1°/s and 5.3°/s for no pF, pF 0.75 and pF 0.65, respectively. In the multi-shot scans, the minimum prostate angular velocities at which signal loss could occur were 16.8°/s, 9.1°/s and 5.6°/s for no pF, pF 0.75 and pF 0.65, respectively. In all cases, the minimum angular velocity at which signal loss could occur decreased with decreasing pF factor, indicating that lower pF values increased the potential sensitivity to motion.

**Fig. 3:**
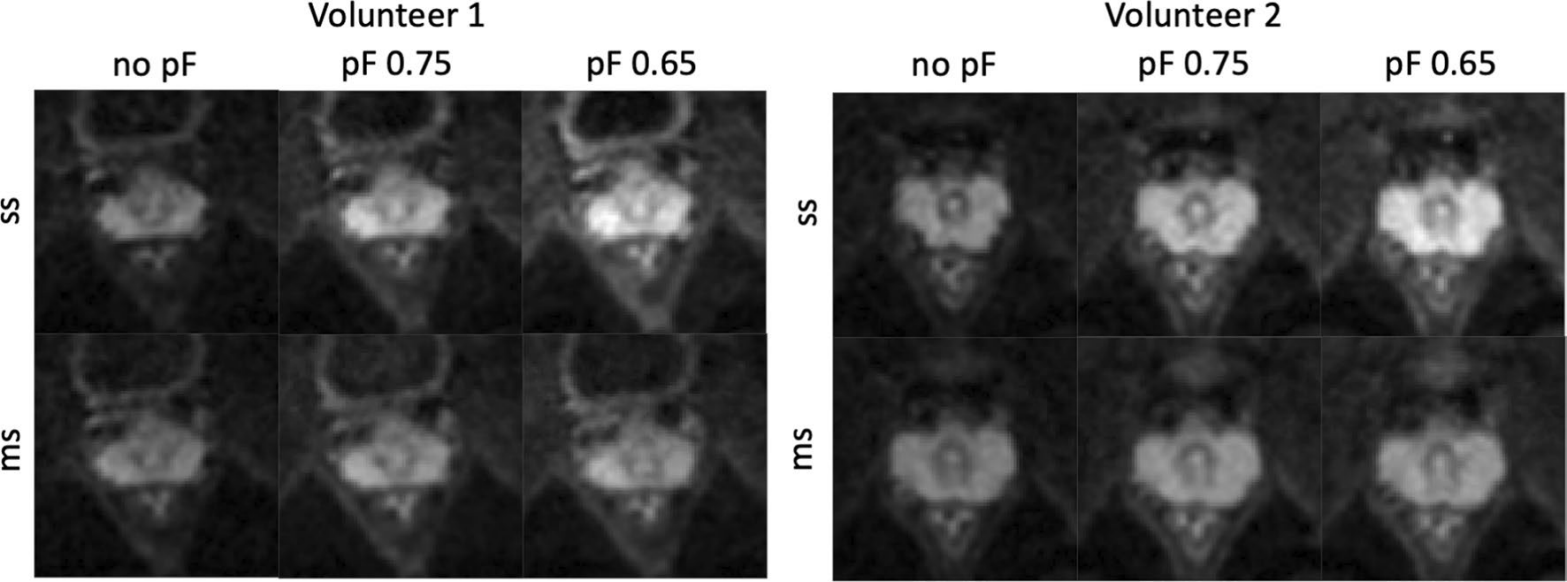
In vivo images of 2 volunteers with different partial Fourier factors for single-shot and multi-shot acquisitions at *b* = 800 s/mm^2^, averaged over repetitions and diffusion directions. All images within each volunteer have the same window level and have been cropped for better visualization. As the partial Fourier factor decreases, the signal intensity increases due to the decrease in echo time

Figure 3 shows in vivo *b* = 800 s/mm^2^ single-shot and multi-shot DWIs for all pF factors and diffusion encoding waveforms. In general, for both volunteers shown, as the pF factor decreased, the signal increased due to the corresponding decrease in TE. Since the images shown in Fig. 3 are averaged images, signs of motion artefacts are not likely to be visible unless they are severe and occur frequently.

**Fig. 4:**
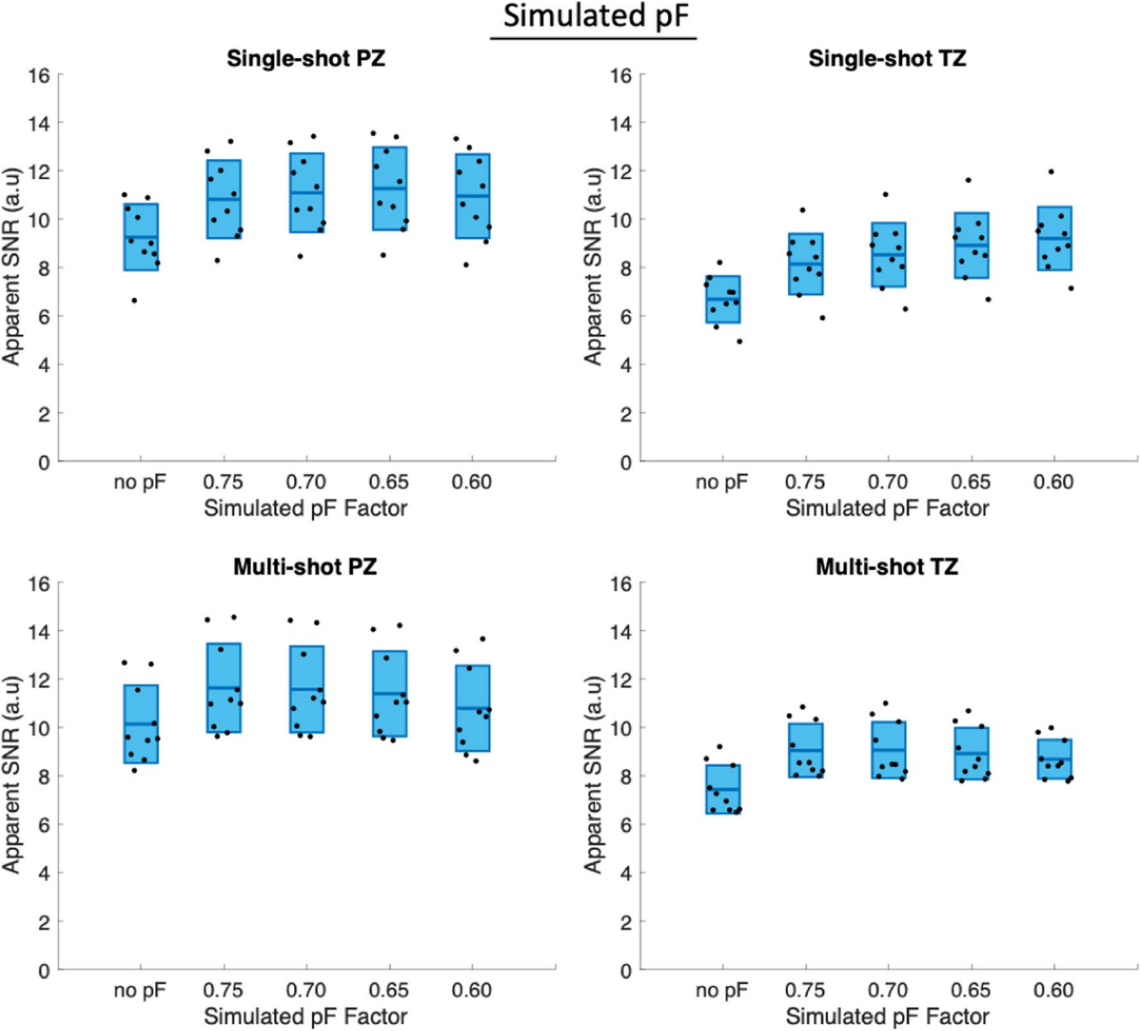
Apparent SNR with simulated pF factors and adjustment of signal level based on TE. In the PZ, both single-shot and multi-shot show an initial increase in apparent SNR, followed by a reduction in apparent SNR as the pF factor decreases. The pF factor at which the apparent SNR reduces is different between single-shot and multi-shot. The TZ used a different *T*_2_ value for the TE adjustment, and therefore gives different results

Figure 4 shows the apparent SNR values of the simulated pF data averaged over diffusion directions, which was simulated from the data acquired without pF encoding and included simulation of the decreased TE in pF acquisitions. The same values are additionally shown in Supplementary Material Table S1. In the single-shot PZ, the apparent SNR increases with decreasing pF factor up to a pF of 0.65, after which there is a slight decrease in apparent SNR. In the single-shot TZ, the apparent SNR increases with decreasing pF for all simulated pF factors, likely as a result of having a shorter *T*_2_ value in the TZ in comparison to the PZ. In the multi-shot case, the apparent SNR increased between no pF and simulated pF 0.75. In the PZ, the apparent SNR then decreased with decreasing pF factor after a pF of 0.75, whereas in the TZ, the apparent SNR increased by a small amount between simulated pF 0.75 and 0.70, then decreased with decreasing simulated pF.

**Fig. 5:**
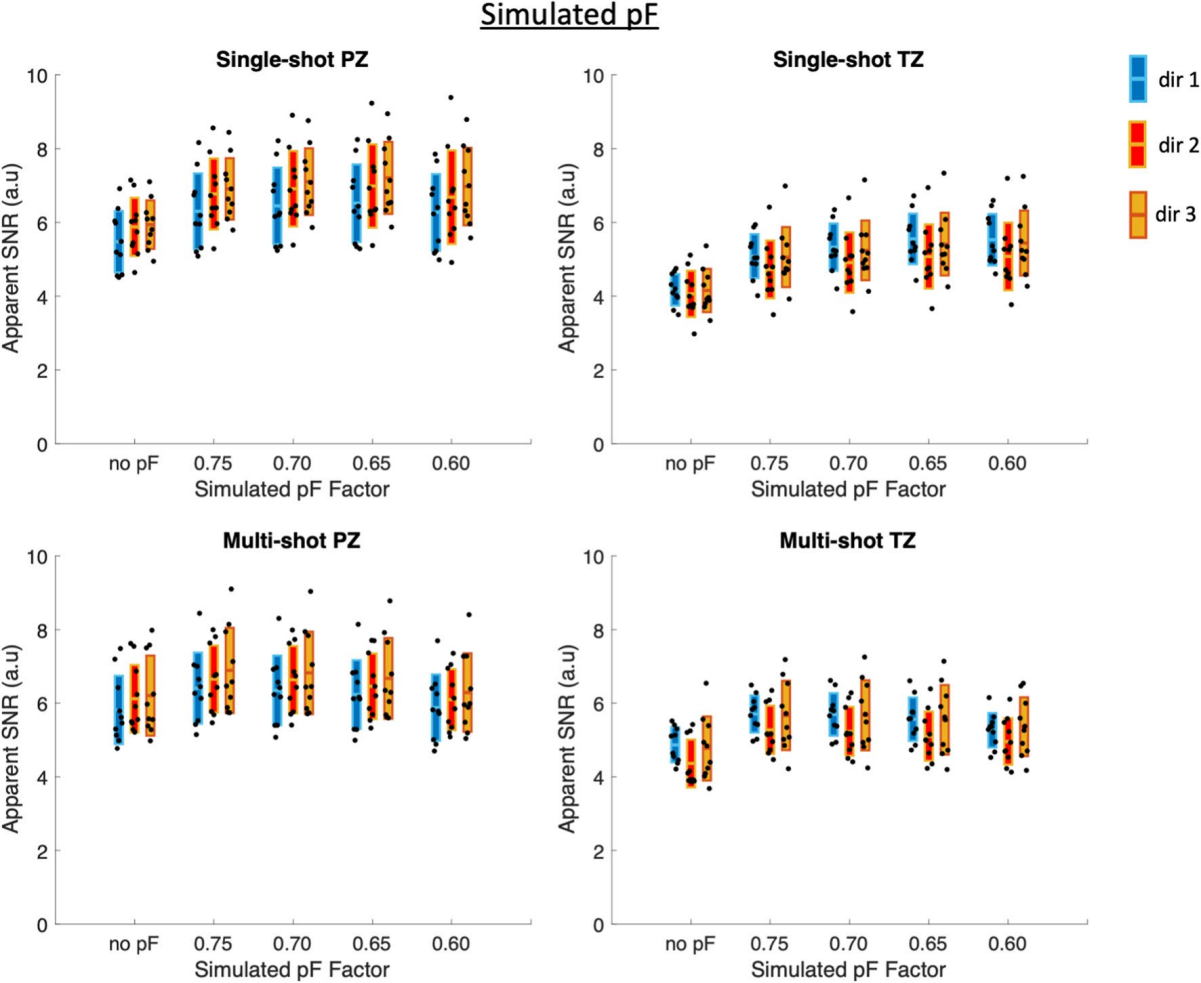
Apparent SNR for each individual diffusion direction with simulated pF factors and adjustment of signal level based on TE. The overall trend is similar to that of the averaged directions, as shown in Fig. 4. Although there are differences in apparent SNR between the diffusion directions, the differences are small

Figure 5 shows the apparent SNR values of the simulated pF data for each individual diffusion direction. The same values are additionally shown in Supplementary Material Table S2. In general, the apparent SNR increases with decreasing simulated pF until pF 0.65 in the single-shot PZ case, and until pF 0.75 in the multi-shot PZ case, after which the apparent SNR decreases. In the single-shot TZ case, the apparent SNR in general increases with decreasing simulated pF factor for all pF factors, apart from dir 1. In the multi-shot TZ case, the apparent SNR increases between no pF and pF 0.75, but then decreases with decreasing pF for dir 1 and dir 2. In general, there are small differences in apparent SNR between all diffusion directions.

**Fig. 6:**
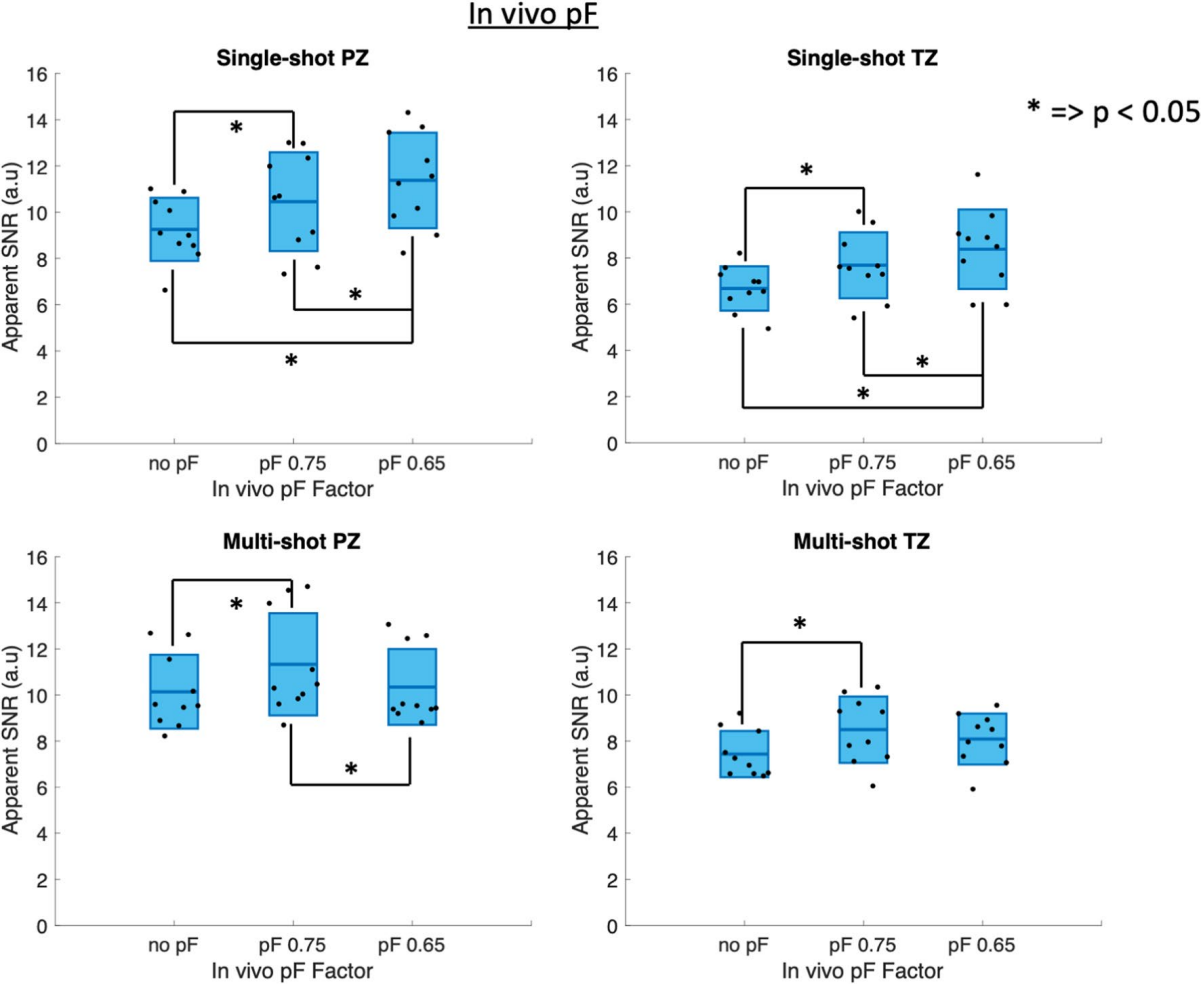
In vivo apparent SNR for single-shot, multi-shot and different pF factors in the peripheral zone (PZ) and transitional zone (TZ). The statistically significant results (*p* < 0.05) are marked with an asterisk (*). In the single-shot PZ case, the apparent SNR increases with decreasing pF factor, indicating that decreasing the pF factor increases the signal due to the shorter echo time whilst still being robust against motion effects. In the multi-shot PZ and TZ cases, both diffusion encoding waveforms showed an increase in apparent SNR between no pF and pF 0.75 but showed an apparent SNR decrease between pF 0.75 and 0.65, suggesting that the small decrease in TE between pF 0.75 and 0.65 was not large enough to counteract the increased sensitivity to motion and reduced SNR from a shorter readout

Figure 6 shows the apparent SNR values of the in vivo acquired pF data for single-shot and multi-shot acquisitions, with statistically significant differences (*p* < 0.05) marked graphically with an asterisk (*). The same values are additionally shown in Supplementary Material Table S3. The *p*-values are shown in Supplementary Material Table S4. Notably, single-shot no pF had significantly lower apparent SNR than all other single-shot scans. Single-shot pF 0.65 had significantly higher apparent SNR than single-shot no pF and single-shot 0.75 pF. The paired t-test results of single-shot pF 0.65 in the PZ suggest that using a pF factor of 0.65 is not too low at a *b*-value of *b* = 800 s/mm^2^ in prostate DWI, as the shorter TE compared to the other single-shot scans caused in increase in apparent SNR. In the multi-shot PZ case, multi-shot pF 0.75 had significantly higher apparent SNR than all other multi-shot scans, suggesting that in multi-shot scans, the marginally shorter TE of multi-shot pF 0.65 compared to multi-shot pF 0.75 does not lead to an increase in apparent SNR, but does potentially sensitise the signal to motion and might increase the signal variability. The results were similar in the TZ to those of the PZ, however, in the TZ the notable differences were that the difference in apparent SNR between multi-shot pF 0.75 and both multi-shot pF 0.65 was no longer statistically significant.

**Fig. 7:**
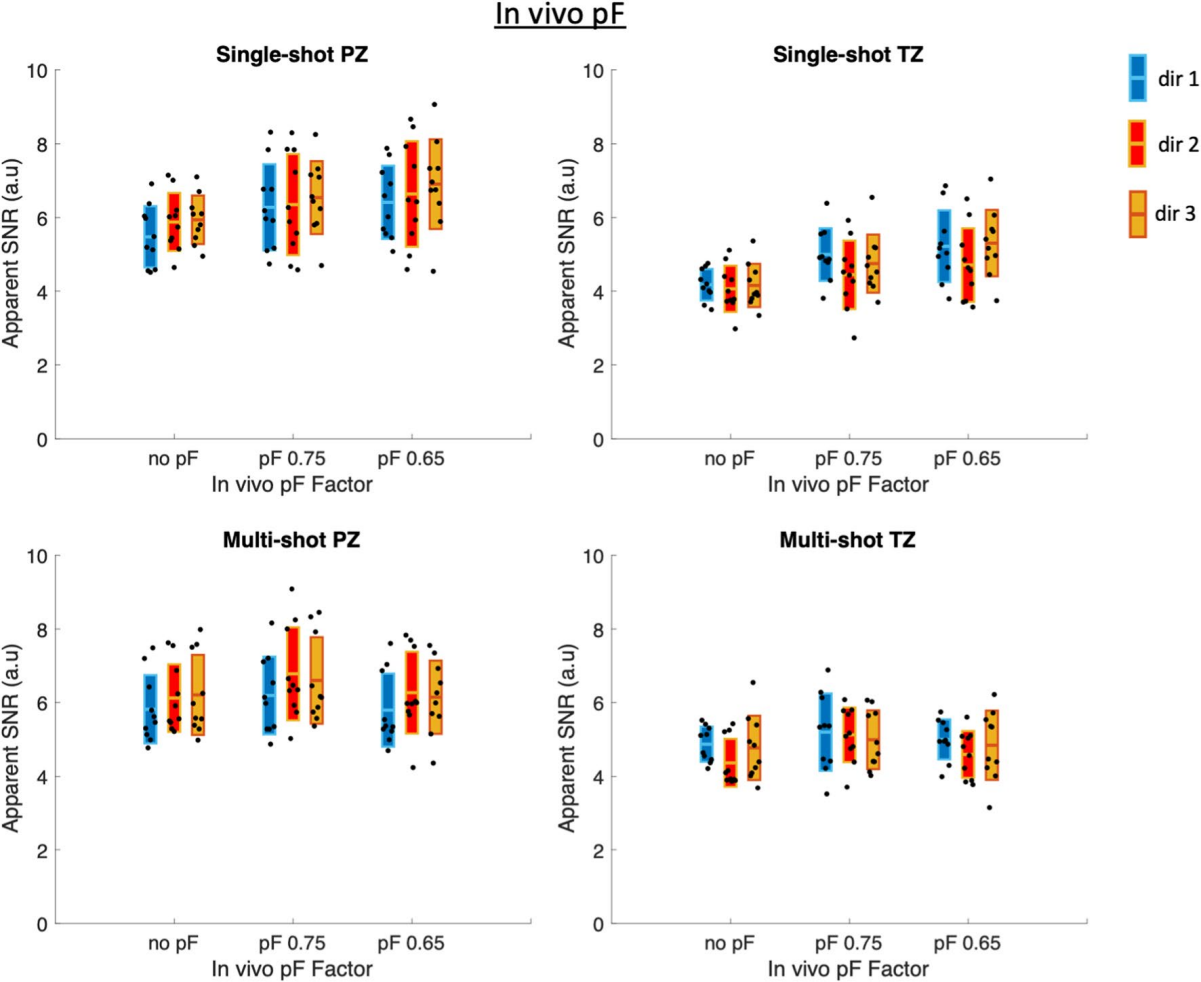
In vivo apparent SNR for single-shot, multi-shot and different pF factors for each diffusion direction in the peripheral zone (PZ) and transitional zone (TZ). The values are additionally shown in Supplementary Material Table S5, and the *p*-value tables are shown in Supplementary Material Tables S6–S12. The general trend is similar to that of the averaged directions shown in Fig. 6. Some of the differences between diffusion directions are statistically significant, however, they are small

Figure 7 shows the apparent SNR values for each individual diffusion direction of the in vivo acquired pF data for single-shot and multi-shot acquisitions. The same values are additionally shown in Supplementary Material Table S5, and the *p*-values are shown in Supplementary Material Tables S6–S12. In general, the apparent SNR values increase with decreasing pF factor in the single-shot acquisitions in the PZ and TZ. In the multi-shot case, the apparent SNR increased between no pF and pF 0.75, but then decreased between pF 0.75 and 0.65 in both the PZ and TZ for all diffusion directions. Some of the differences in apparent SNR between diffusion directions are statistically significant. Notably, the apparent SNR for dir 2 does not increase as much as dir 1 and dir 3 in the single-shot TZ case.

**Fig. 8:**
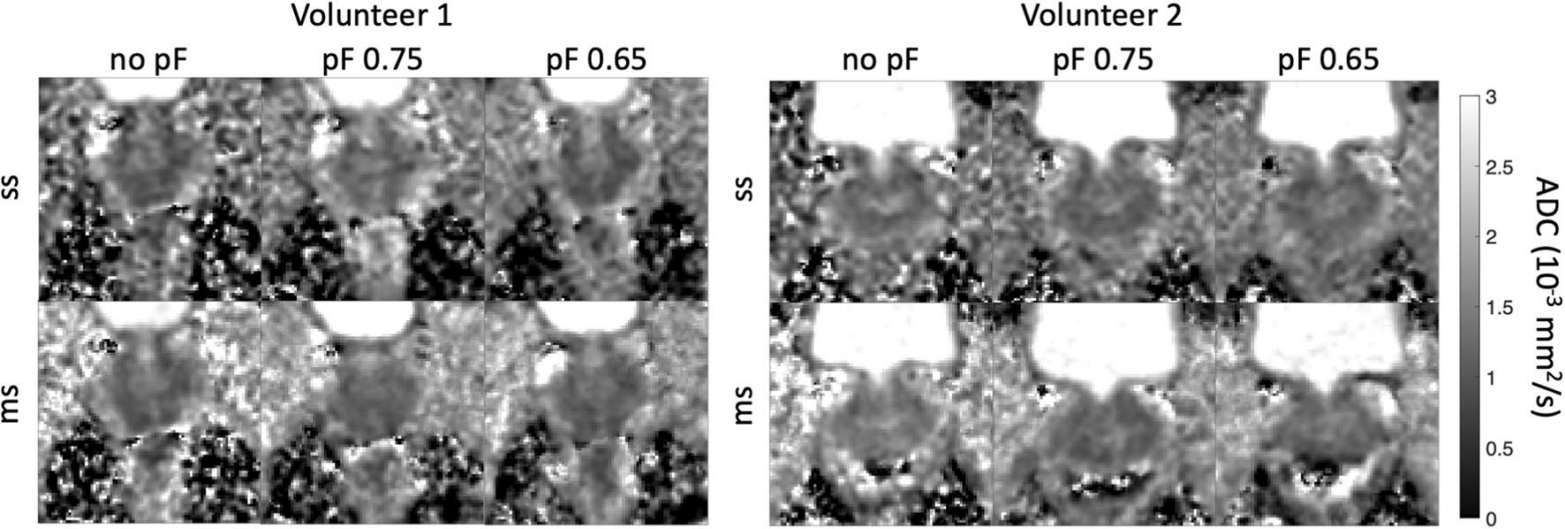
Cropped FOV ADC maps of 2 volunteers with different partial Fourier factors for single-shot and multi-shot acquisitions to show the comparable quality between the pF factors and between the number of shots. Note that the 2 volunteers are different to the 2 volunteers shown in the DWIs in Fig. 6

**Fig. 9:**
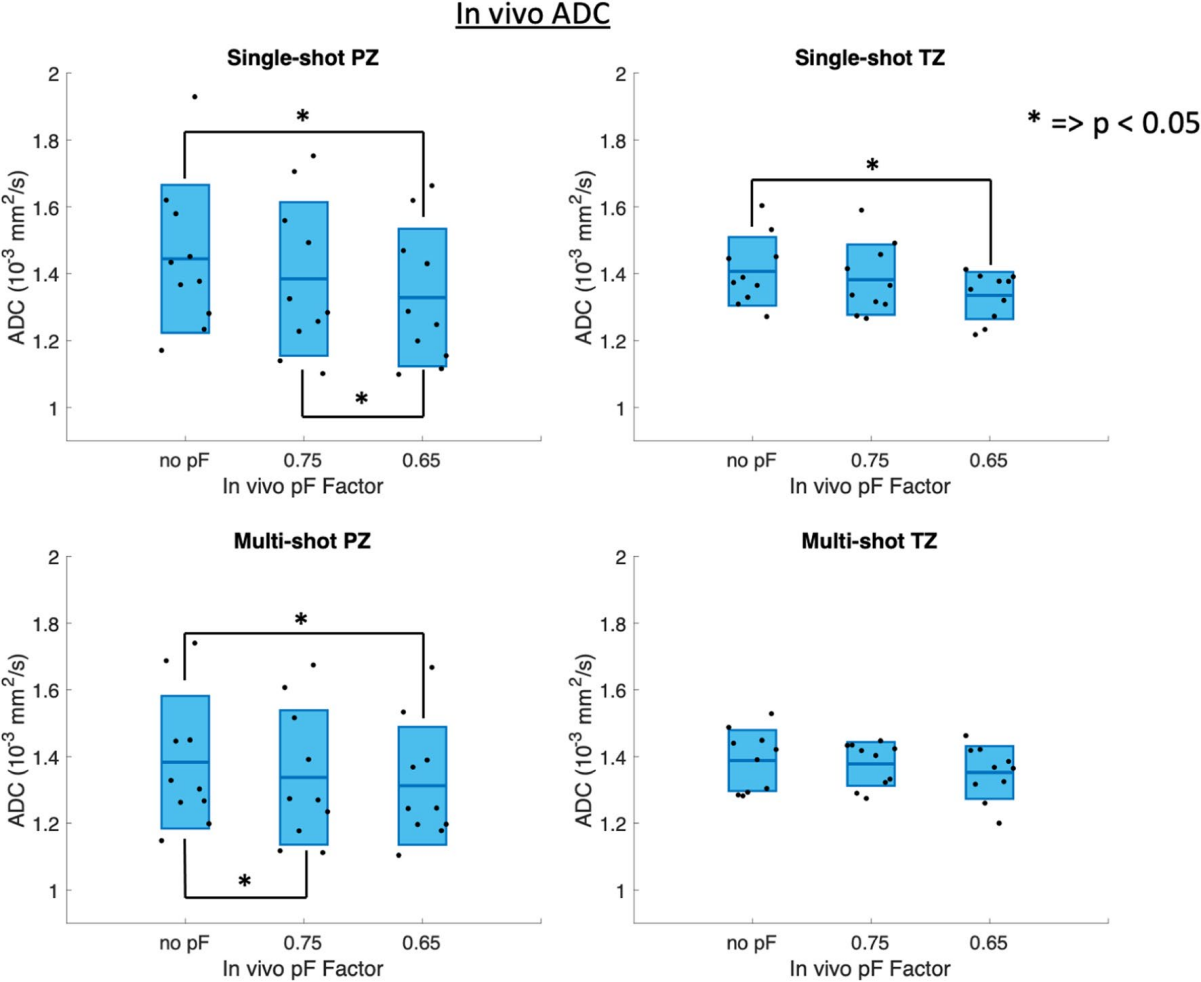
In vivo ADC values for single-shot, multi-shot and different pF factors in the peripheral zone (PZ) and transitional zone (TZ). The statistically significant results (*p* < 0.05) are marked with an asterisk (*). In general, as the pF factor decreases, the ADC value also decreases. Since intravoxel dephasing due to motion is more likely to cause an increase in ADC, the decrease in ADC with decreasing pF factor can be caused by *T*_2_ effects at shorter echo times

Figure 8 shows the ADC maps for all acquisitions. Figure 9 shows the ADC values of the in vivo acquired pF data, for both diffusion encoding waveforms and for single-shot and multi-shot acquisitions, with statistically significant differences (*p* < 0.05) marked graphically with an asterisk (*). The same ADC values are additionally shown in Supplementary Material Table S13, and the *p*-values are given in Supplementary Material Table S14. Notably, the reduction in ADC in the PZ for single-shot pF 0.65 in comparison to single-shot pF 0.75 was significant. In the multi-shot case, the difference in ADC between multi-shot no pF and multi-shot pF 0.75, as well as the difference in ADC between multi-shot pF 0.75 and multi-shot pF 0.65 was statistically significant. The ADC value results indicate a small but statistically significant relationship between TE and ADC value. Notably, the ADC of single-shot no pF was significantly higher than that of single-shot 0.65 pF.

The PSF simulation shown in Supplementary Material Fig. S2 shows that the loss in effective resolution from a non-moving source due to the homodyne reconstruction is minimal.

## Discussion

The present work assesses the impact of respiratory motion on prostate DWI and characterises the usage of partial Fourier in both single-shot and multi-shot acquisitions. Respiratory motion effects are presently shown as a potential source of intravoxel dephasing in prostate DWI, mostly in the case of deep breathing patterns. Despite potentially increasing the susceptibility to phase-related motion artefacts, pF acquisitions decrease the echo time by reducing the number of acquired *k*-space lines in an EPI readout, which can, therefore, increase the SNR of diffusion weighted acquisitions [14, 15]. The present work showed that in single-shot acquisitions, prostate DWI was still robust with pF factors as low as 0.65, whereas in multi-shot acquisitions, prostate DWI was only robust with pF factors as low as 0.75. Although using low pF factors can increase the apparent SNR as a result of reduced TE, motion can cause the *k*-space centre to shift and cause signal loss or reduce the effective resolution [15].

Partial Fourier has been used in the DWI of other organs. Storey et al. [15] used an adaptive homodyne algorithm to reduce artefacts caused by rigid body motion in the brain. Chang et al. [17] extended the use of an adaptive homodyne algorithm to multi-shot acquisitions in the brain. An adaptive homodyne algorithm was not used in the present work and could be the subject of further study. Zhang et al. [18] were able to correct for severe motion artefacts with motion compensated waveforms in the liver whilst using a pF factor of 0.75. Geng et al. [19] also showed severe motion artefacts which were corrected with motion compensated waveforms in the pancreas, and also used a pF factor of 0.75. Van et al. [16] showed that phase correction can reduce motion-induced worm like artefacts and signal loss in a homodyne reconstruction in the liver. A similar phase correction could be used in the prostate, however, the severity of motion in the prostate is typically lower than in the liver.

Zhang et al. [43] have used a reduced FOV (rFOV) as a method to reduce geometric distortions and directly compared rFOV acquisitions to multi-shot acquisitions in prostate DWI. Although both methods gave good reproducibility of quantitative metrics such as ADC, rFOV had lower SNR than multi-shot, whereas multi-shot had a longer scan time.

The controlled-breathing dynamic scans with simulated pF showed that respiratory motion can also cause intravoxel dephasing in the prostate in cases of deep breathing, which is exacerbated by decreasing the pF factor. Supplementary Material Fig. S1 shows many cases of severe signal loss in the deep breathing case, suggesting that an increase in signal CV was more due to these multiple instances of severe signal loss rather than misregistration effects. The multi-shot acquisitions typically showed lower signal variation than the single-shot acquisitions, which may be due to the averaging effect of combining multiple shots that were acquired during different phases of respiration.

In the free-breathing DWI pF scan results, the averaged DWIs showed increased signal intensity due to the decreased echo time of the pF acquisitions. The simulated pF results typically showed an initial increase in apparent SNR with decreasing pF, followed by a decrease in apparent SNR. The pF factor at which the reduction in apparent SNR occurred was different between single-shot and multi-shot acquisitions as a result of the differences in TEs. The decrease in apparent SNR with decrease in pF factor can occur due to the *T*_2_ associated increase in signal being counteracted by the reduced SNR of a shorter readout in pF acquisitions, as well as a potential increase in the likelihood of motion related artefacts due to the partial *k*-space acquisition when using pF. The apparent SNR measurements of the acquired in vivo pF scans gave a measure of signal variability. Although a shorter TE would increase the signal level and therefore apparent SNR, an increased sensitivity to motion or reduced readout length could cause a lower apparent SNR. The apparent SNR measurements of the acquired in vivo pF scans agreed reasonably well with the findings of the simulated pF results in the single-shot case, as the decreased echo time of the pF 0.65 resulted in an increase in apparent SNR. In the multi-shot case, the in vivo pF 0.75 had significantly (*p* < 0.05) higher SNR than the in vivo pF 0.65 scan in the PZ, which also agreed well with the simulation.

When the apparent SNR maps were split into the individual diffusion directions, the simulated pF results in general agreed well with the in vivo pF results. Although some of the differences between diffusion directions were statistically significant, the differences were small and could have been due to isotropic diffusion giving less signal overall in some diffusion directions. There was no strong evidence to suggest that certain diffusion directions are more prone to signal loss artefacts from motion, and the overall trends of the individual diffusion directions were similar to the data averaged over all diffusion directions.

The ADC maps showed that the ADC values between all waveforms, pF factors and number of shots were similar. However, there were small but statistically significant differences between the ADC values of some of the scans. As motion effects are more likely to cause an increase in ADC, and decreasing the pF factor makes the acquisition more prone to motion artefacts, the observed differences in ADC are less likely to be caused by motion. The observed differences in ADC are more likely to be caused by the reduced echo time [71, 72]. Previous works have shown that long echo times emphasize long *T*_2_ signals in prostatic glands and these signals are characterized by a high ADC [71, 72]. Therefore, as the pF factor decreases, the TE decreases and thus the ADC would be expected to decrease.

In Zhang et al. [38], there were two scans with an in plane resolution of 1.6 × 1.6 mm^2^ in a single-shot acquisition, but one scan had no pF and the other had a pF factor of 0.75. The pF 0.75 scan showed an increase in SNR, which agrees well with our presented results. Zhang et al., however, did not use another pF factor below 0.75 and did not perform multi-shot acquisitions. The ENCODE diffusion encoding waveform in Zhang et al. used an asymmetric design with additional eddy current compensation. As the ENCODE waveform was able to use a full Fourier acquisition without a large difference in TE in comparison to using pF, the ENCODE waveform without pF was less sensitive to motion artefacts such as signal loss and a loss in effective resolution [38]. The present work showed that in the absence of motion, the effect of pF on the effective resolution is minimal. Other works have used pF in the prostate and did not report any negative effects of employing pF [38, 45, 61–65].

The present work has some limitations. First, only 10 healthy volunteers were scanned, with a subset of 5 volunteers for the controlled breathing dynamic scans, due to the long scan times making it impractical to scan prostate cancer patients. However, even with only 10 volunteers, statistically significant differences (*p* < 0.05) were found between some of the scans. Second, no bowel preparation was performed on the volunteers prior to the scans. If there was less bowel motion, then it is possible that multi-shot pF 0.65 would have had higher apparent SNR the multi-shot pF 0.75. Third, as there was some prostate motion between scans and ROIs were adjusted accordingly, differences in the ROIs could have affected the results. However, given that the ROIs were drawn over most of the PZ and TZ, and also averaged over 3 slices, any small differences in the ROIs would most likely have been averaged out. Fourth, the present work used DWI acquired at two *b*-values and 3 diffusion directions and thus did not model perfusion effects and diffusion anisotropy effects. The employed lower *b*-value of 100 s/mm^2^ was chosen to reduce the effect of perfusion [9, 73], however, it is possible that some perfusion signal remained, which can confound the ADC values. Fifth, although the controlled-breathing dynamic scans showed that prostate DWI can be affected by intravoxel dephasing caused by breathing motion, the volunteers were told to breathe deeply, which is not how someone would normally breathe and is, therefore, not directly applicable to a standard free-breathing DWI scan. However, the purpose of the experiment was to show that breathing induced intravoxel dephasing in prostate DWI is possible, and to show the effect of using different simulated pF factors. Sixth, eddy current effects were not presently addressed. However, the presented arguments should hold in general for waveform designs that minimize eddy current effects [37, 38]. Seventh, the diffusion encoding directions were different between the controlled breathing dynamic scans and the free-breathing DWI pF scans, which could mean that the results from each experiment are not directly applicable to each other, as the signal loss pattern is determined by the interaction between motion and diffusion encoding direction. However, three orthogonal diffusion directions were measured in the free-breathing DWI pF scans, meaning that directional dependence on susceptibility to motion would be captured, although it would also be averaged with the other directions. Eighth, due to scan time constraints, only one phase encoding direction was chosen for each experiment. Ninth, the simulation of the pF factors cannot exactly simulate the acquired pF scans as it was done by removing *k*-space lines from a scan that was acquired without pF. As the pF factor only applies to the readout of the imaging echo and not the navigator echo, the multi-shot scans were simulated with the same navigator for all simulated pF factors. It is, therefore, possible that the multi-shot scans were more affected by the discrepancies between the simulated and acquired in vivo pF scans. The literature *T*_2_ values used in the simulation were the same for all volunteers, as *T*_2_ mapping was not performed in the present work. Tenth, the *p*-values were not adjusted for multiple comparisons between tests [74]. Finally, the presented reported conclusions in the use of certain pF factors depend on the employed *b*-value. The use of pF encoding in prostate DWI at higher *b*-values should be addressed in future works.

## Conclusion

Respiratory motion effects were assessed, and the usage of different pF factors were characterized in the context of prostate DWI in both single-shot and multi-shot acquisitions. The controlled-breathing dynamic scans showed that breathing motion can cause intravoxel dephasing and therefore signal loss in cases of deep breathing patterns, which can be even more severe with lower pF factors. However, in the low pF free-breathing pF DWI single-shot acquisitions at a clinically relevant *b*-value of 800 s/mm^2^ (which did not have deep breathing patterns), any risks of motion related artefacts were outweighed by the increase in signal from a lower TE, as pF 0.65 had the highest apparent SNR. In multi-shot acquisitions however, the highest apparent SNR was given by a pF factor of 0.75, showing that using lower pF factors in prostate DWI may not be worthwhile if the reduction in TE is not large enough. In addition, a reduction in TE can also cause a small reduction in the measured ADC value.

## Supplementary Information

Below is the link to the electronic supplementary material.

Supplementary file 1 (DOCX 1720 kb)

Supplementary file 2 (MP4 2151 kb)

Supplementary file 3 (TIFF 4633 kb)

Supplementary file 4 (TIFF 3034 kb)

Supplementary file 5 (TIFF 9054 kb)

Supplementary file 6 (TIFF 2315 kb)

Supplementary file 7 (TIFF 4402 kb)

Supplementary file 8 (TIFF 7949 kb)

Supplementary file 9 (TIFF 3923 kb)

Supplementary file 10 (TIFF 3876 kb)

Supplementary file 11 (TIFF 3881 kb)

Supplementary file 12 (TIFF 4364 kb)

Supplementary file 13 (TIFF 4303 kb)

Supplementary file 14 (TIFF 4218 kb)

Supplementary file 15 (TIFF 4242 kb)

Supplementary file 16 (TIFF 2022 kb)

Supplementary file 17 (TIFF 4155 kb)

## Abbreviations

DWI: Diffusion weighted imaging
ADC: Apparent diffusion coefficient
ss: Single-shot
ms: Multi-shot
EPI: Echo planar imaging
ODGD: Optimized diffusion-weighting gradient waveform design
pgse: Pulsed gradient spin echo
pF: Partial Fourier
