# Supplementary material for: Partial Fourier in the presence of respiratory motion in prostate diffusion-weighted echo planar imaging
Source: MAGMA. 2024 May 14;37(4):621–36. doi: 10.1007/s10334-024-01162-x (PMC11417066; doi:10.1007/s10334-024-01162-x)
Supplement: Supplementary file 1 — (DOCX 1720 kb) [file 10334_2024_1162_MOESM1_ESM.docx]

**CONTROLLED BREATHING DYNAMIC DWI SCANS**

**Fig. S1:** Shown in the video (provided separately in the file Figure_S1_controlled_breathing_deep_shallow_video.mp4) are the single-shot deep and shallow scans from a single subject. The deep breathing case has a few dynamics in which the prostate signal is completely lost, and a few dynamics in which there is signal loss. In the shallow breathing case, the signal level is more stable across dynamics

**Table S1:** Apparent SNR in the peripheral zone (PZ) and transitional zone (TZ) of the prostate over multiple simulated partial Fourier factors for the single-shot and multi-shot acquisitions. The data are taken from Fig. 4.


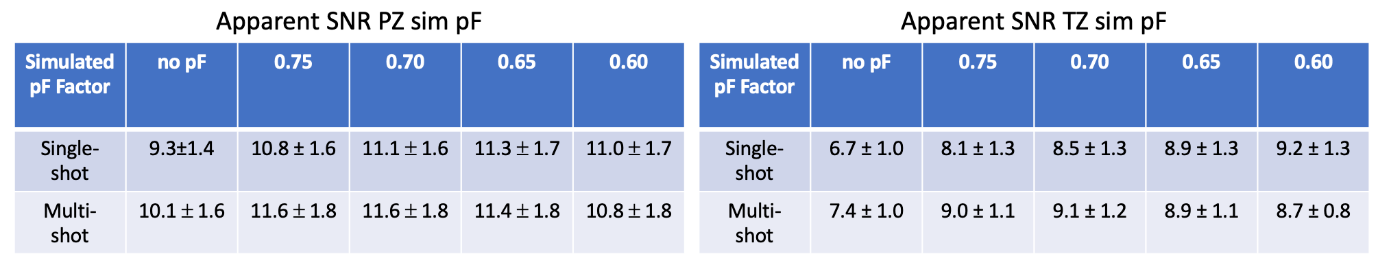


**Table S2:**  Apparent SNR for each diffusion direction in the peripheral zone (PZ) and transitional zone (TZ) of the prostate over multiple simulated partial Fourier factors for the single-shot and multi-shot acquisitions. The data are taken from Fig. 5


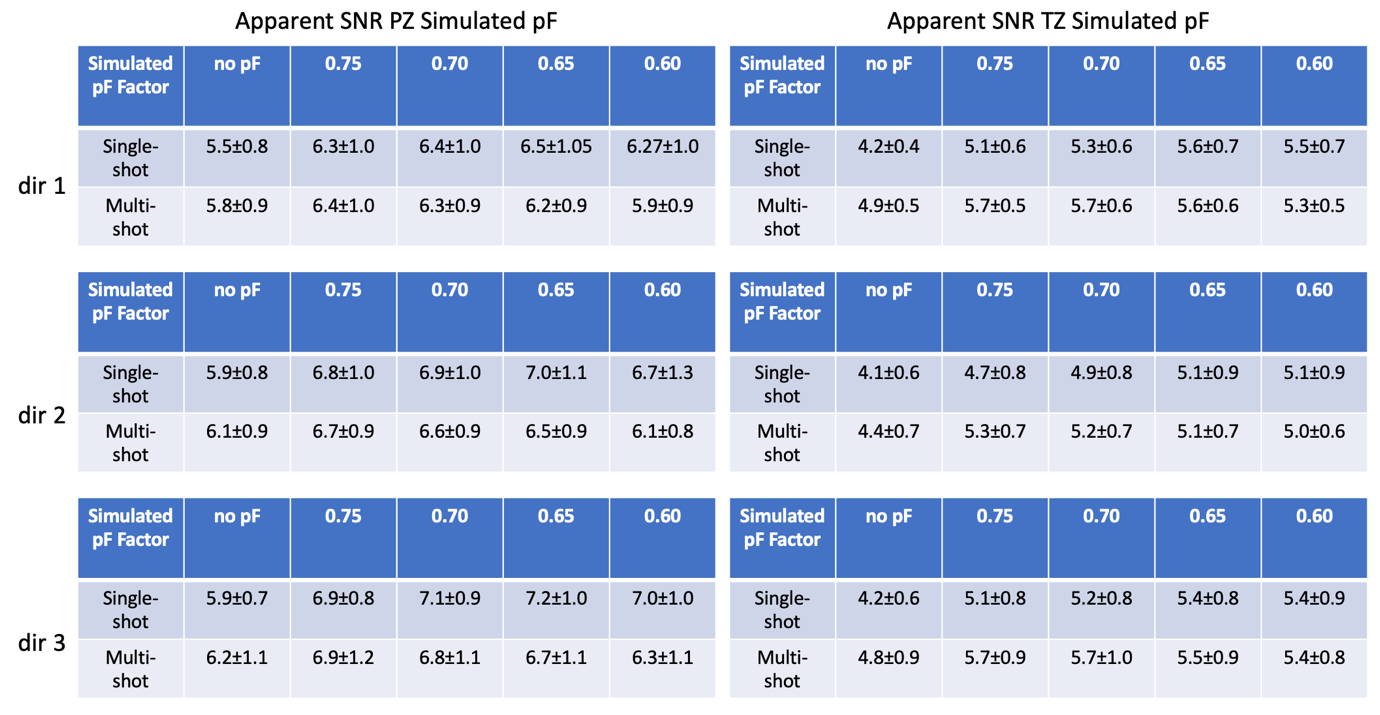


**Table S3:** In vivo apparent SNR in the peripheral zone (PZ) and transitional zone (TZ) of the prostate for the single-shot and multi-shot acquisitions. The data are taken from Fig. 6


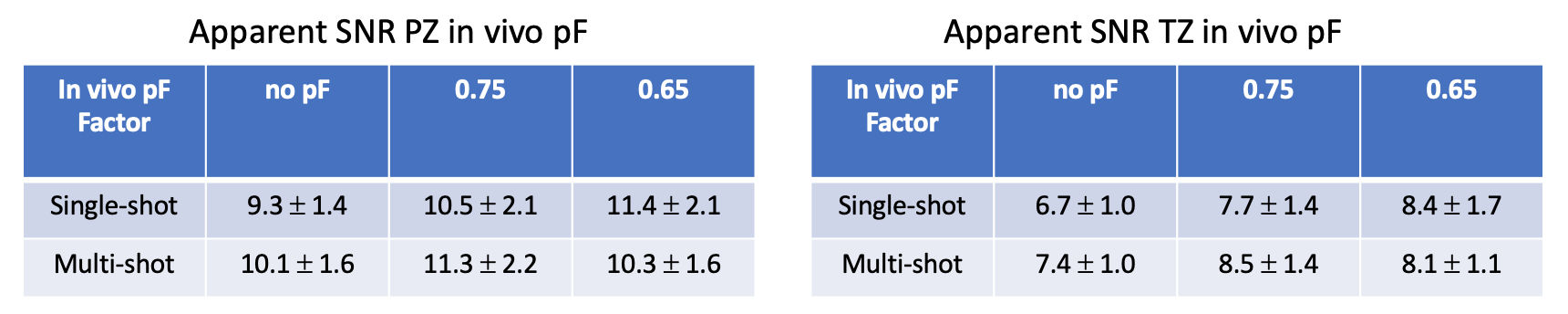


**Table S4:** p-value table for the in vivo apparent SNR values given in Fig. 6. Statistically significant values (p < 0.05) are marked with an asterisk (*).


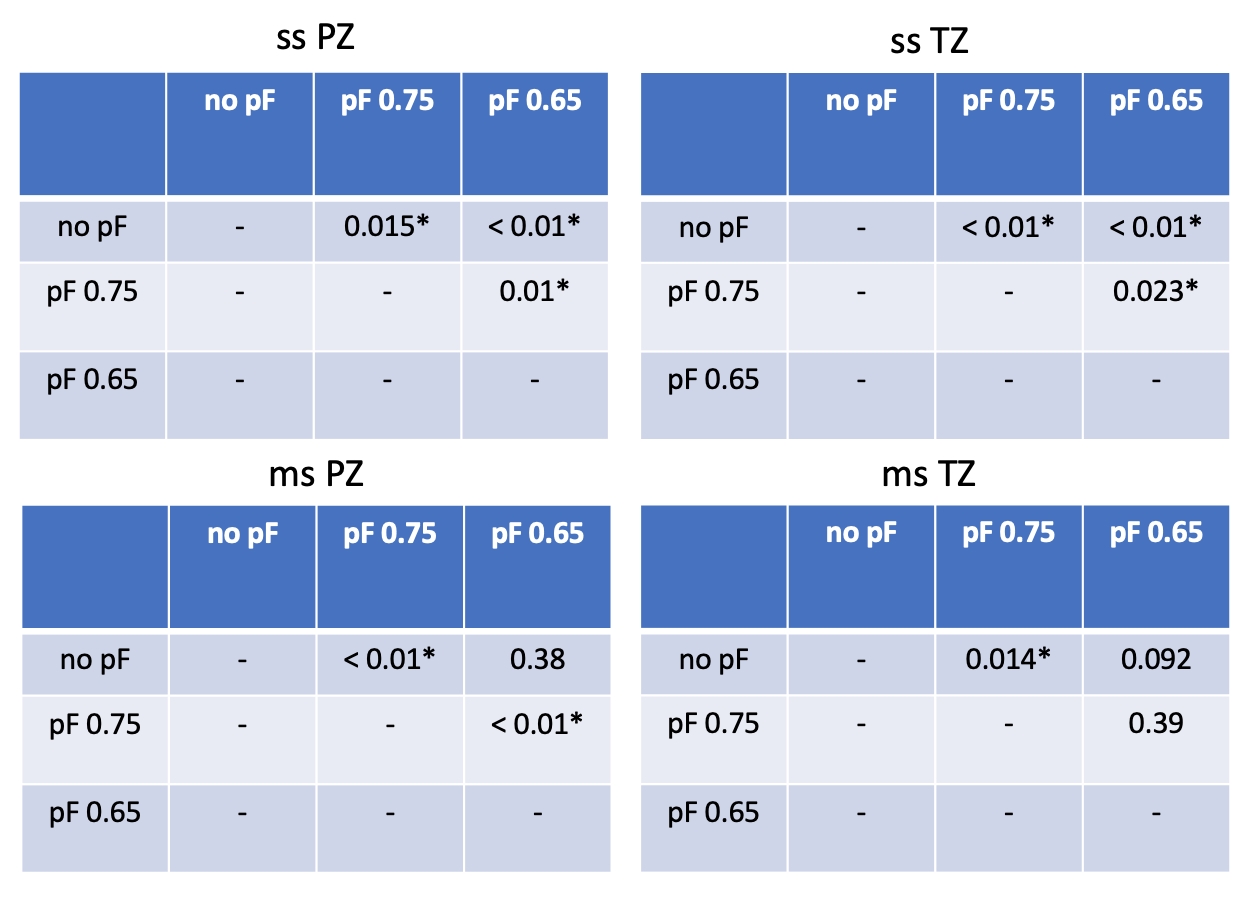


**Table S5:** In vivo apparent SNR for each diffusion direction in the peripheral zone (PZ) and transitional zone (TZ) of the prostate for the single-shot and multi-shot acquisitions. The data are taken from Fig. 7


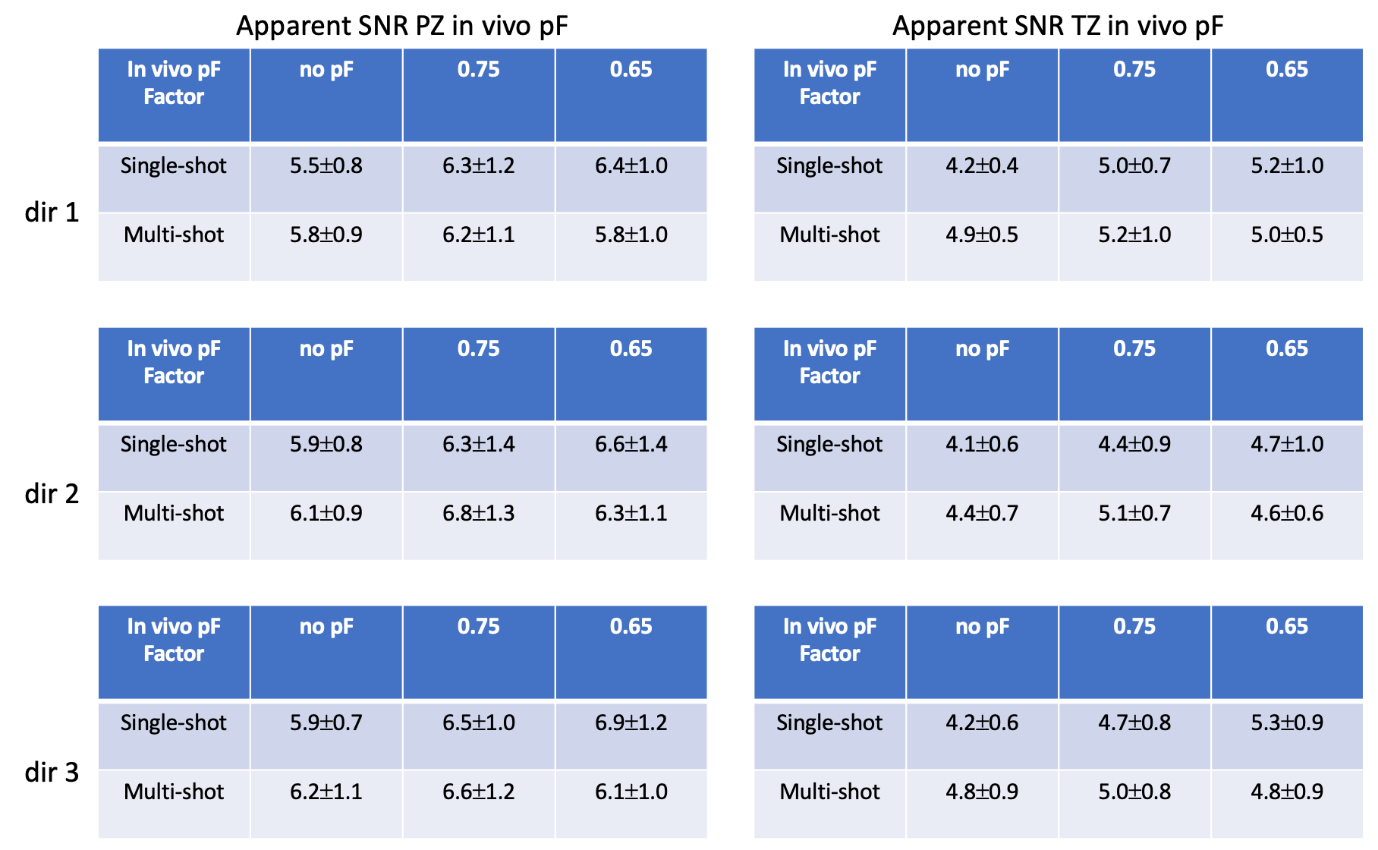


**Table S6:** p-values for dir 1 of the in vivo apparent SNR results per diffusion direction. Statistically significant results (p < 0.05) are marked with an asterisk (*)


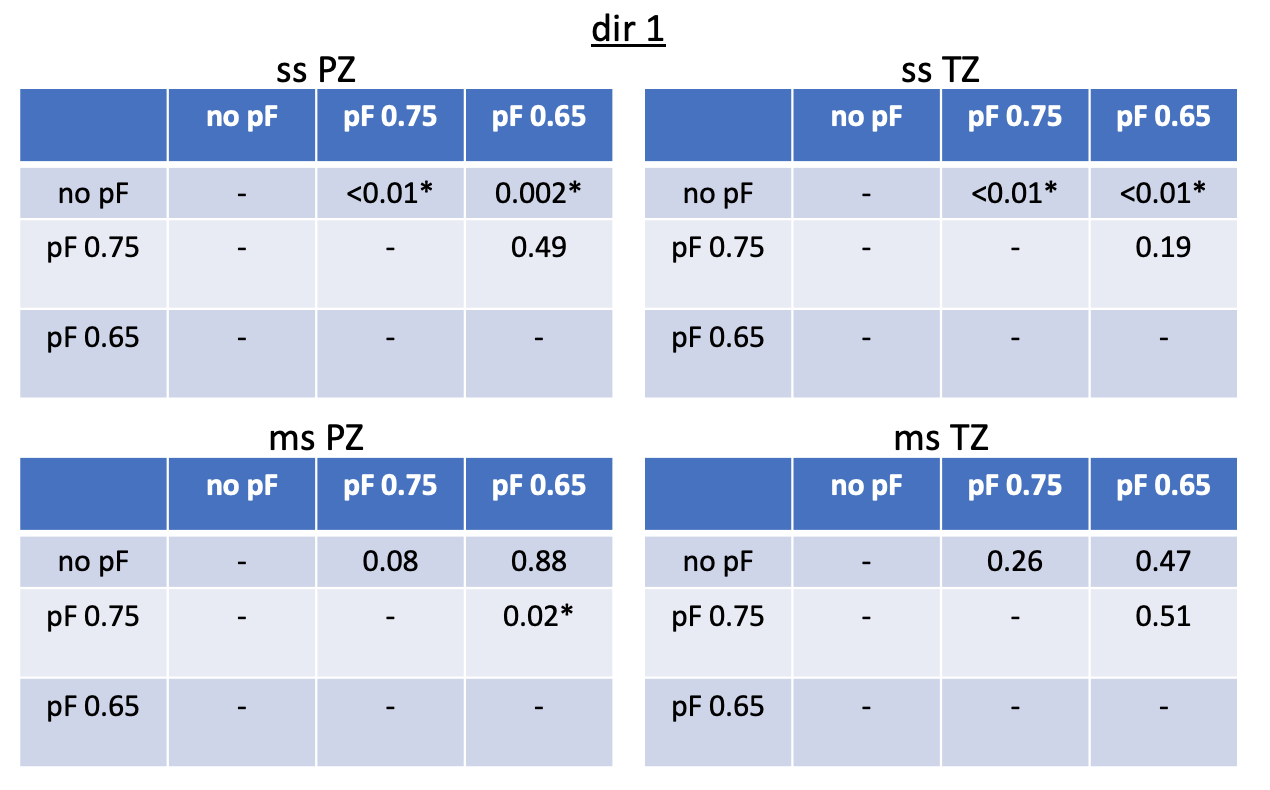


**Table S7:** p-values for dir 2 of the in vivo apparent SNR results per diffusion direction. Statistically significant results (p < 0.05) are marked with an asterisk (*)


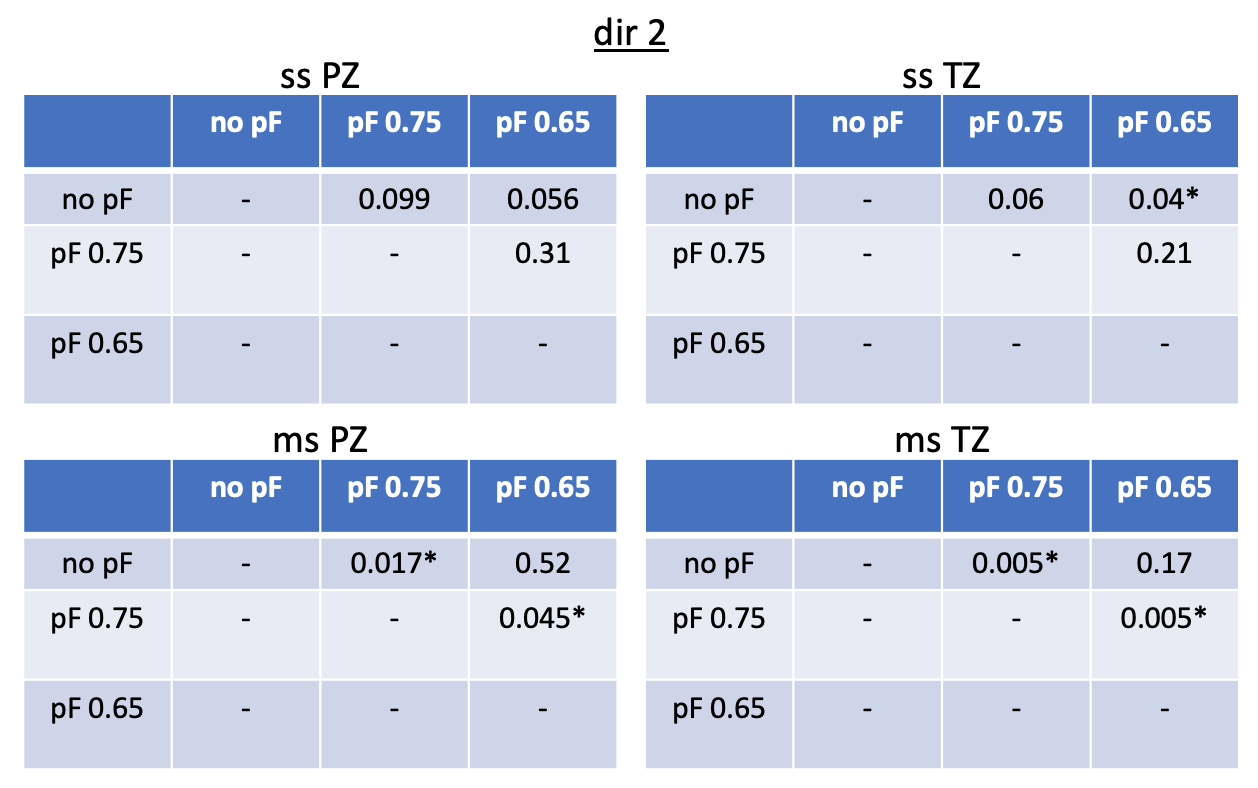


**Table S8:** p-values for dir 3 of the in vivo apparent SNR results per diffusion direction. Statistically significant results (p < 0.05) are marked with an asterisk (*)


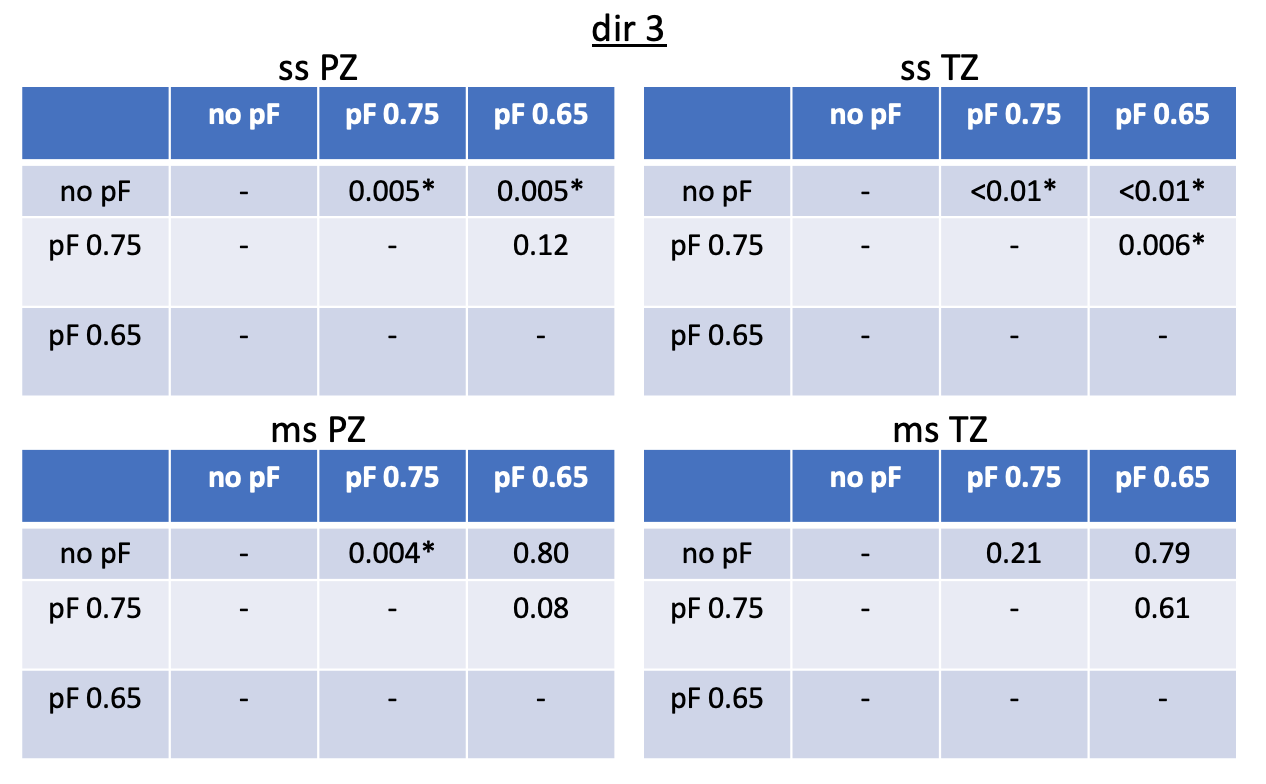


**Table S9:** p-values for the comparisons between diffusion directions in single-shot PZ in vivo apparent SNR results. Statistically significant results (p < 0.05) are marked with an asterisk (*)

**
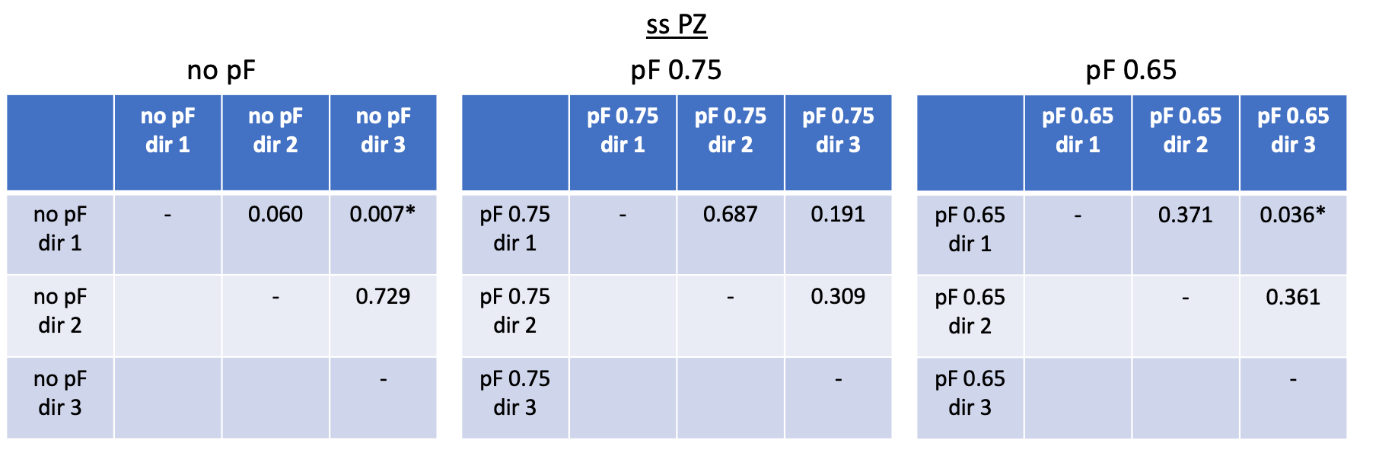
**

**Table S10:** p-values for the comparisons between diffusion directions in single-shot TZ in vivo apparent SNR results. Statistically significant results (p < 0.05) are marked with an asterisk (*)

**
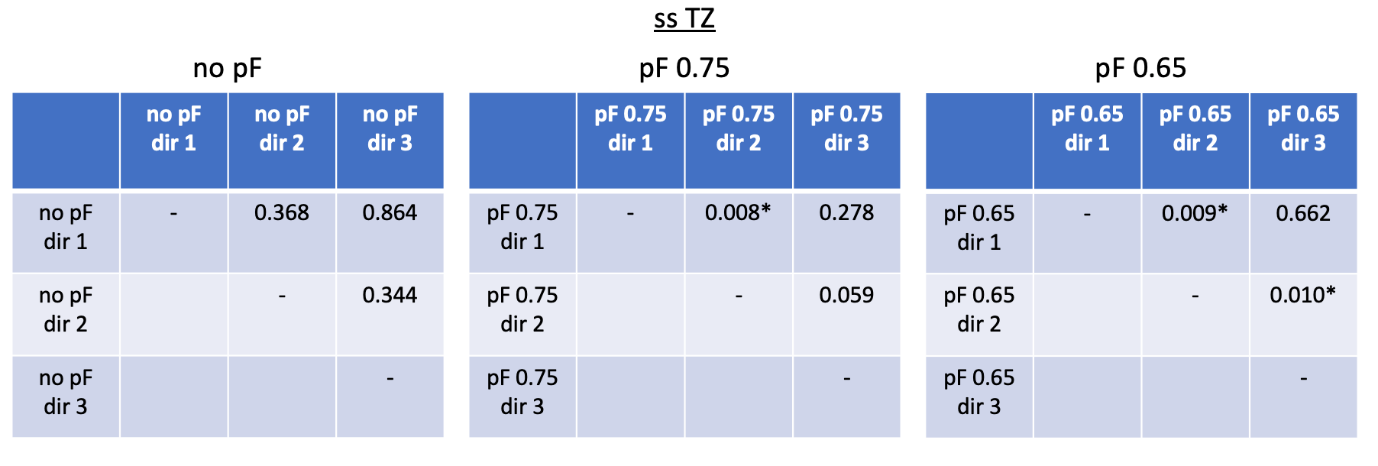
**

**Table S11:** p-values for the comparisons between diffusion directions in multi-shot PZ in vivo apparent SNR results. Statistically significant results (p < 0.05) are marked with an asterisk (*)

**
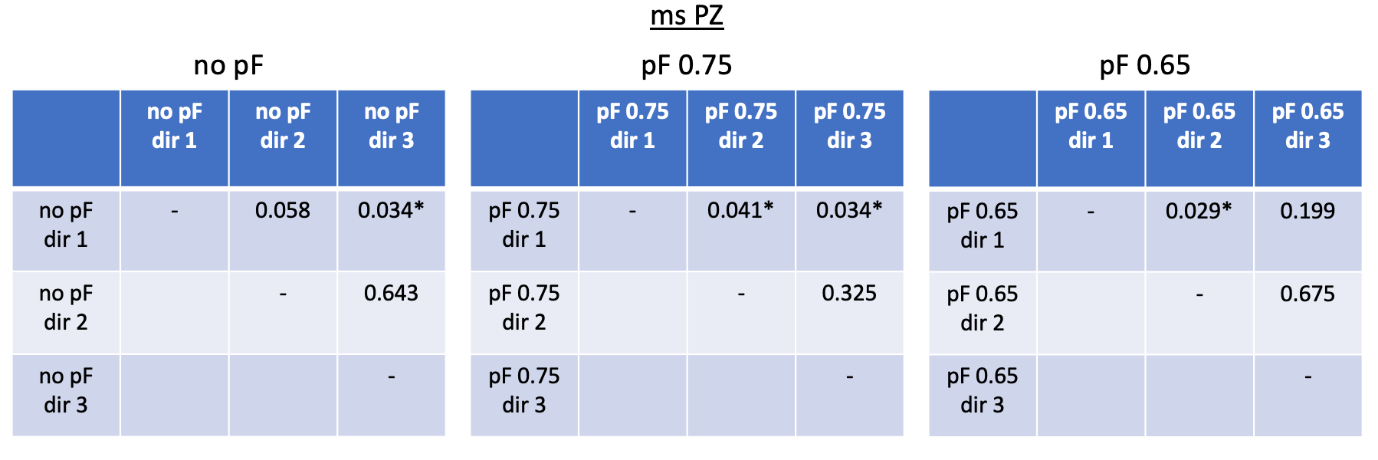
**

**Table S12:** p-values for the comparisons between diffusion directions in multi-shot TZ in vivo apparent SNR results. Statistically significant results (p < 0.05) are marked with an asterisk (*)

**
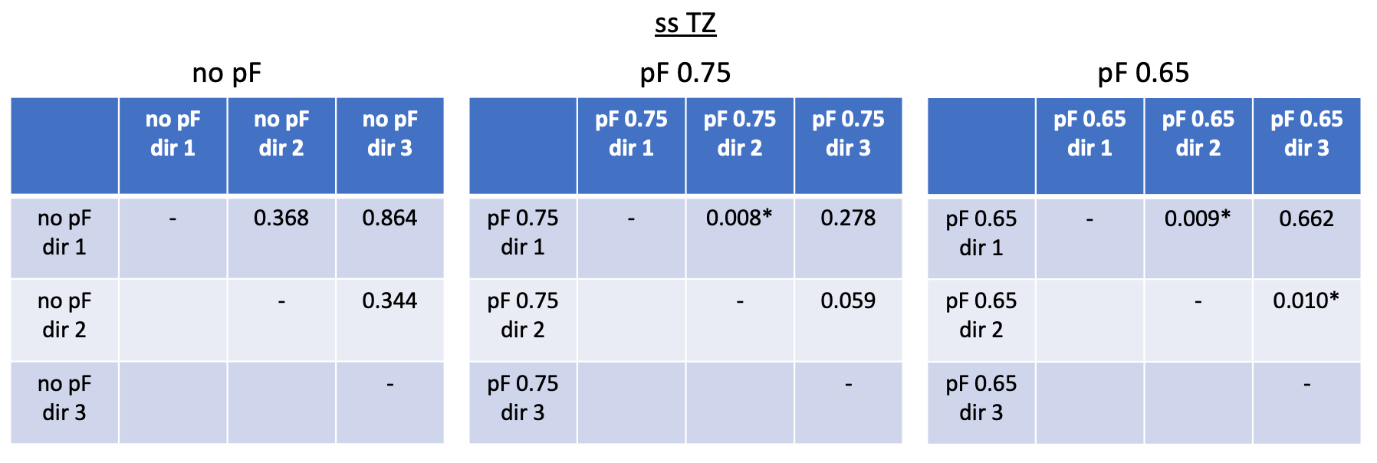
**

**Table S13:** In vivo ADC values. The data are taken from Fig. 9

**
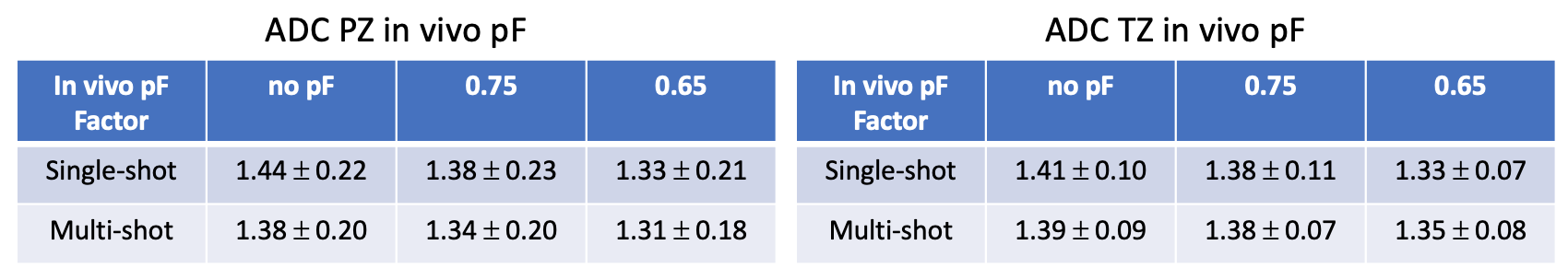
**

**Table S14:** p-value table for the in vivo ADC values. Statistically significant results (p < 0.05) are marked with an asterisk (*)


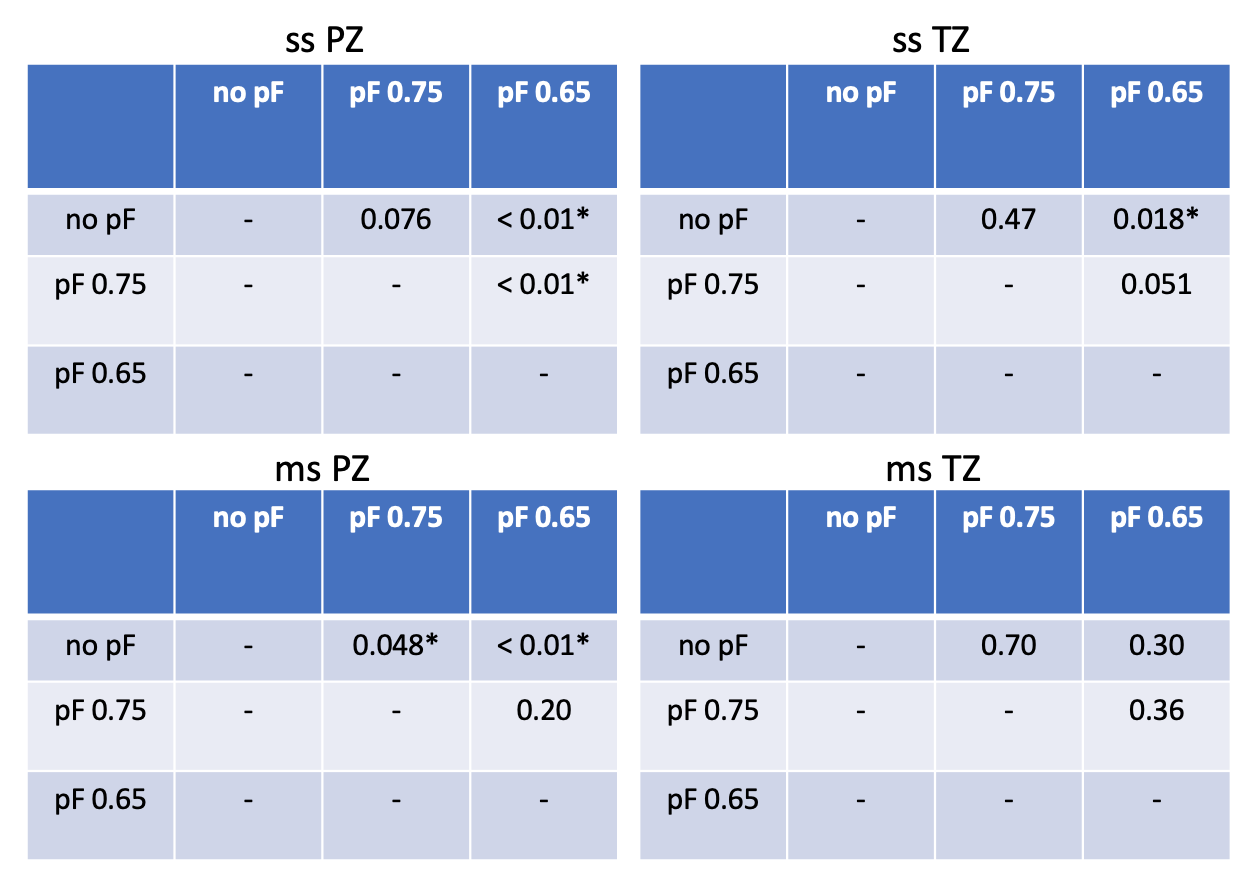


**POINT SPREAD FUNCTION SIMULATION**

**Purpose:** The purpose of the point spread function (PSF) simulation was to simulate the impact of pF on the effective resolution in the absence of motion.

**Method**: A single point source in an array size of 300 x 280, which is the interpolated array size including oversampling of the reconstructed image in the free-breathing DWI partial Fourier scans, was transformed to k-space. The array was then reduced in size according to the pF factor and reconstructed back to image space with a homodyne reconstruction, and the full width half maximum (FWHM) was measured.

**
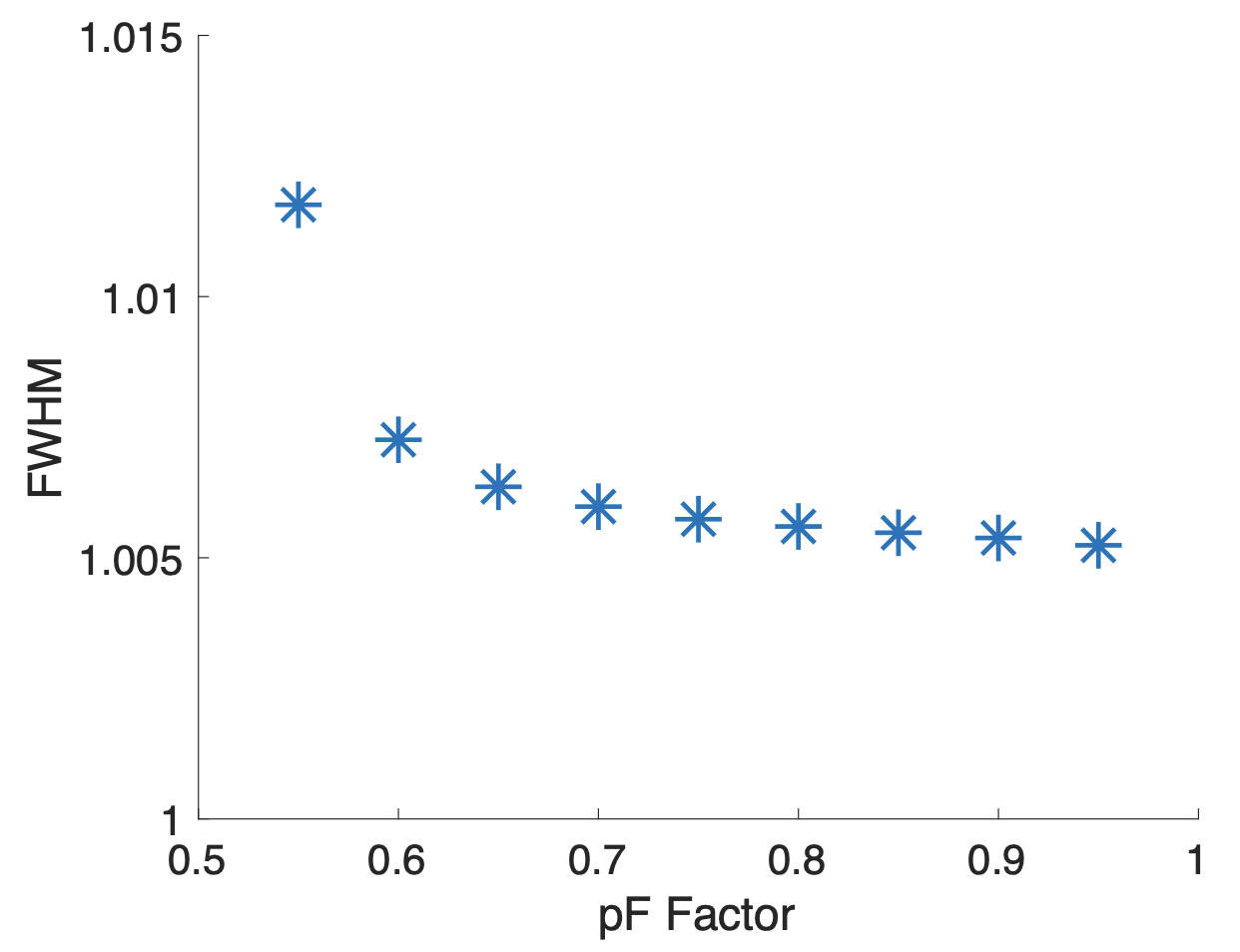
**

**Fig. S2:** FWHM of the pF PSF simulation. The FWHM of no pF is 1. The only pF factor which has a FWHM larger than 1% of the FWHM of the no pF acquisition is 0.55. There is a minimal difference in the FWHM for pF factors larger than 0.55
