# Supplementary figures and images for: Partial Fourier in the presence of respiratory motion in prostate diffusion-weighted echo planar imaging
Source: MAGMA. 2024 May 14;37(4):621–36. doi: 10.1007/s10334-024-01162-x (PMC11417066; doi:10.1007/s10334-024-01162-x)

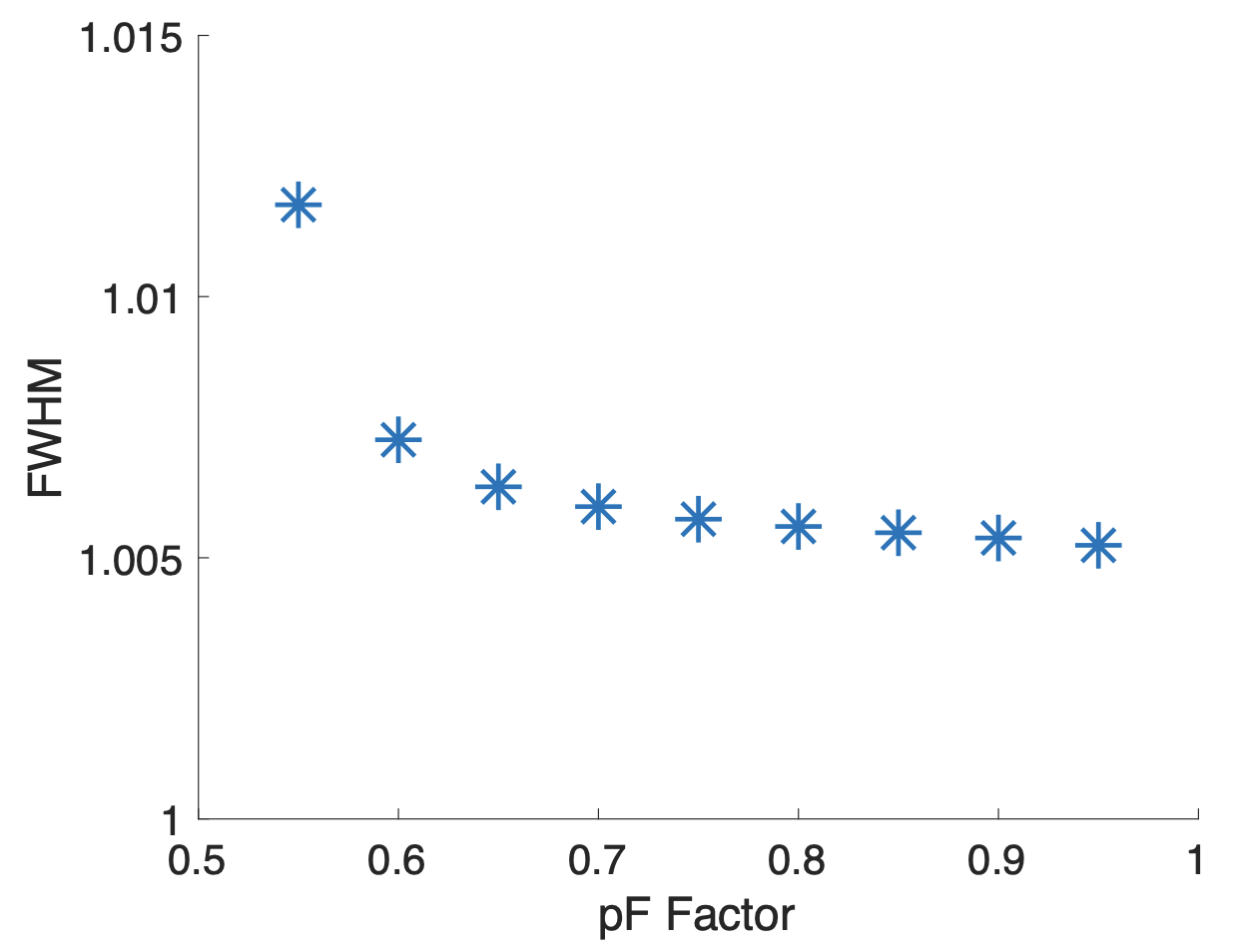

Supplement: Supplementary file 3 — (TIFF 4633 kb) [file 10334_2024_1162_MOESM3_ESM.tiff]

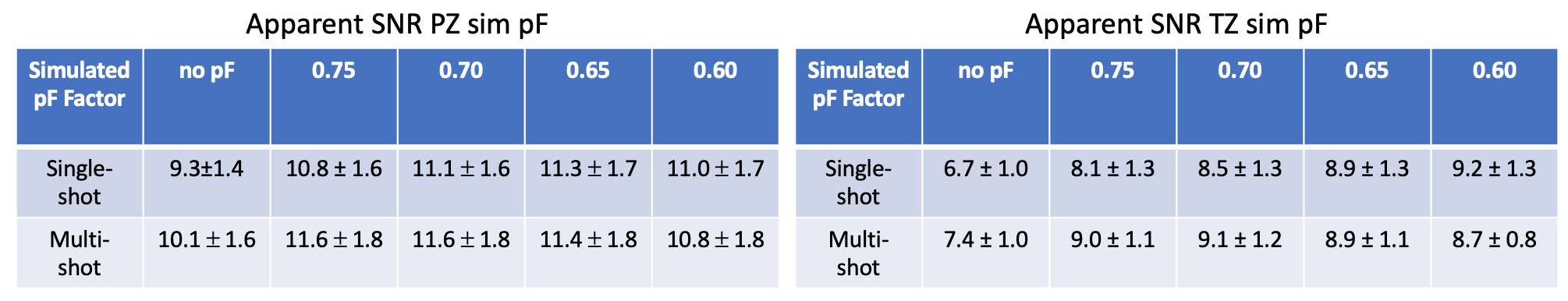

Supplement: Supplementary file 4 — (TIFF 3034 kb) [file 10334_2024_1162_MOESM4_ESM.tiff]

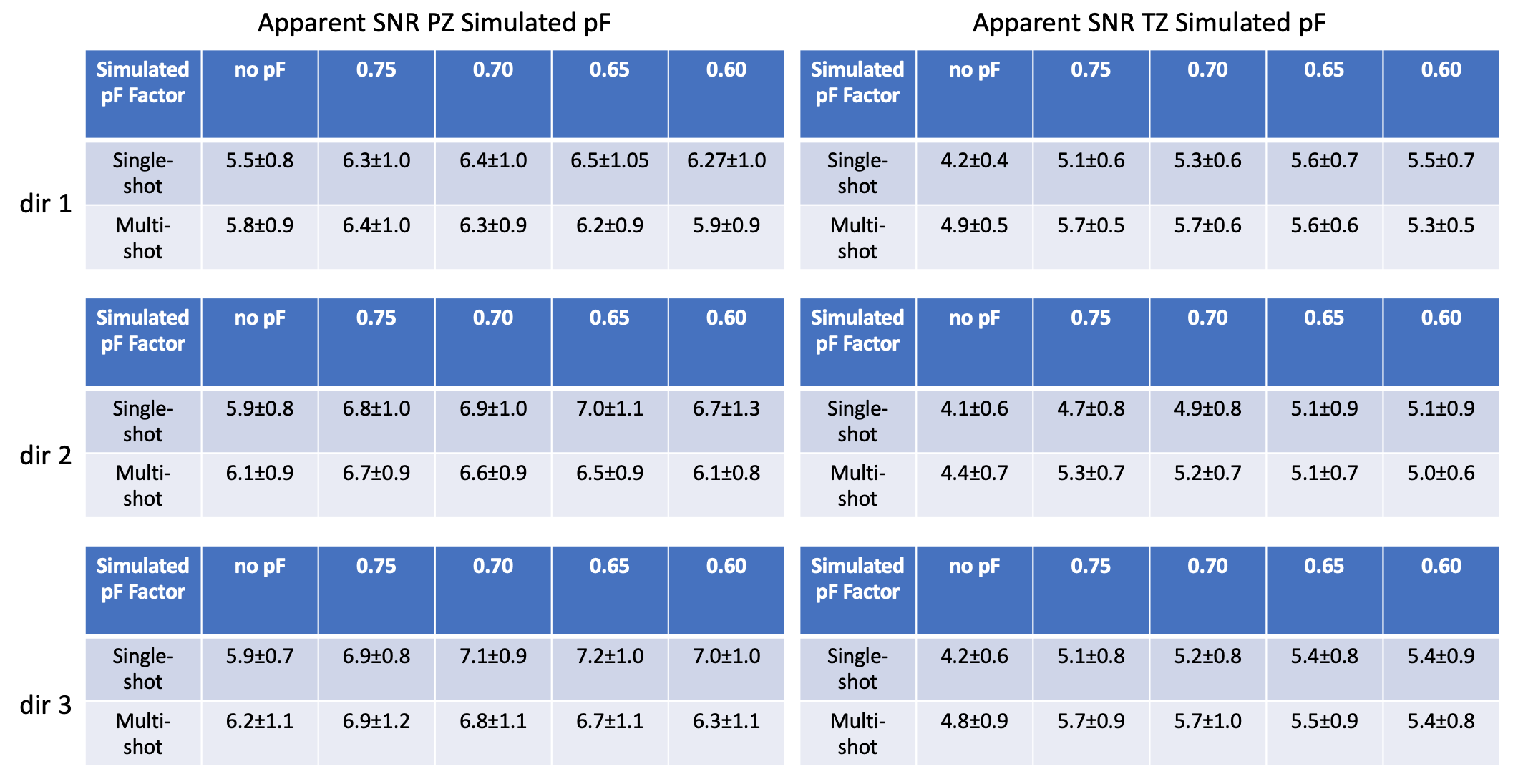

Supplement: Supplementary file 5 — (TIFF 9054 kb) [file 10334_2024_1162_MOESM5_ESM.tiff]

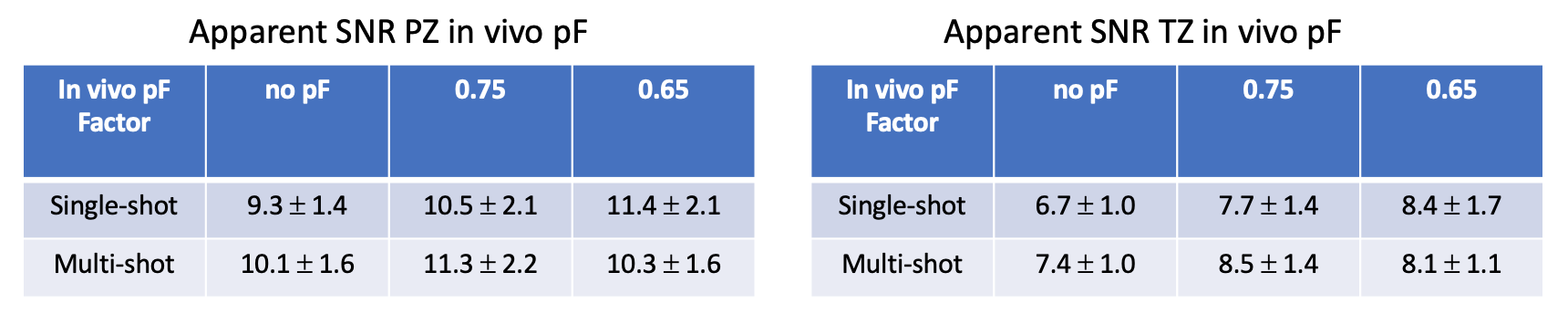

Supplement: Supplementary file 6 — (TIFF 2315 kb) [file 10334_2024_1162_MOESM6_ESM.tiff]

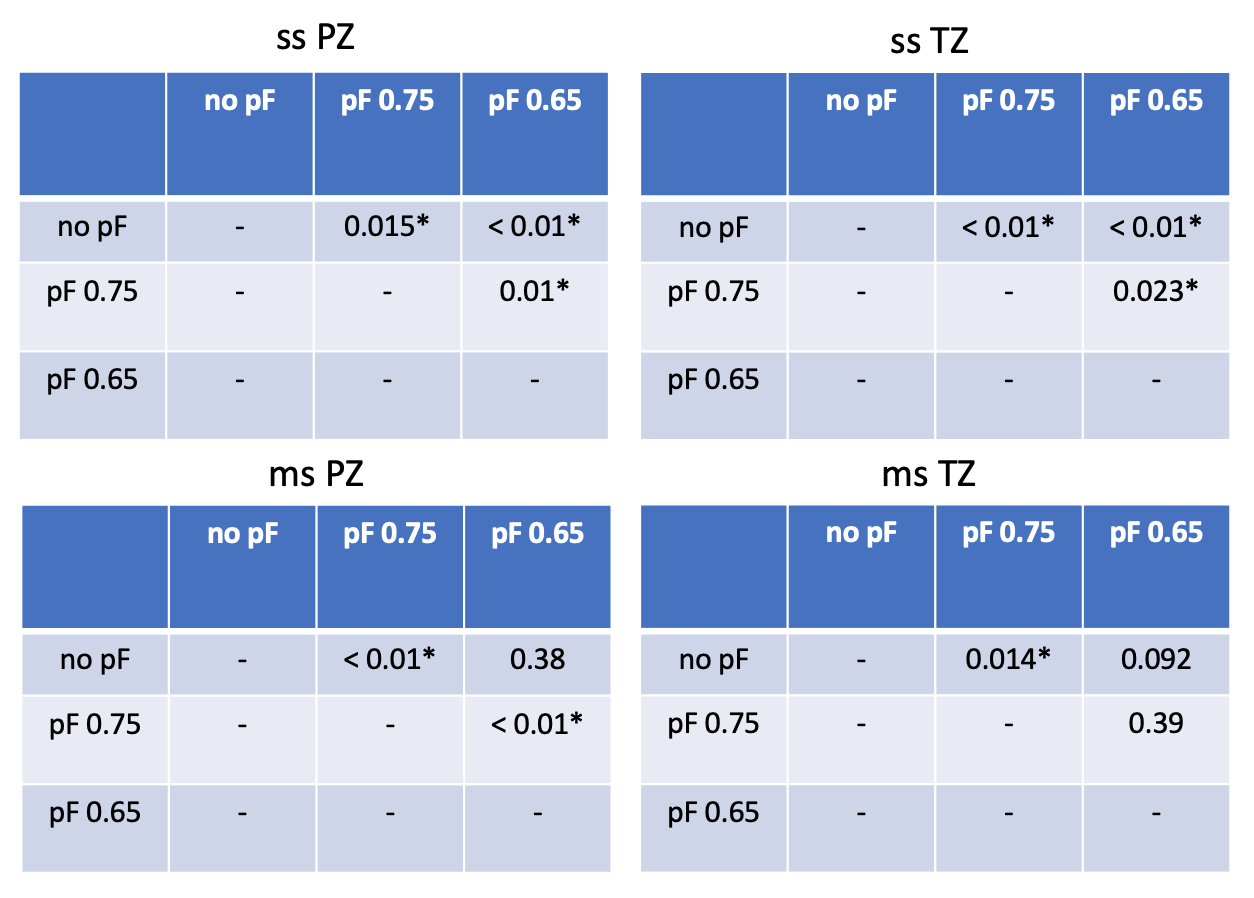

Supplement: Supplementary file 7 — (TIFF 4402 kb) [file 10334_2024_1162_MOESM7_ESM.tiff]

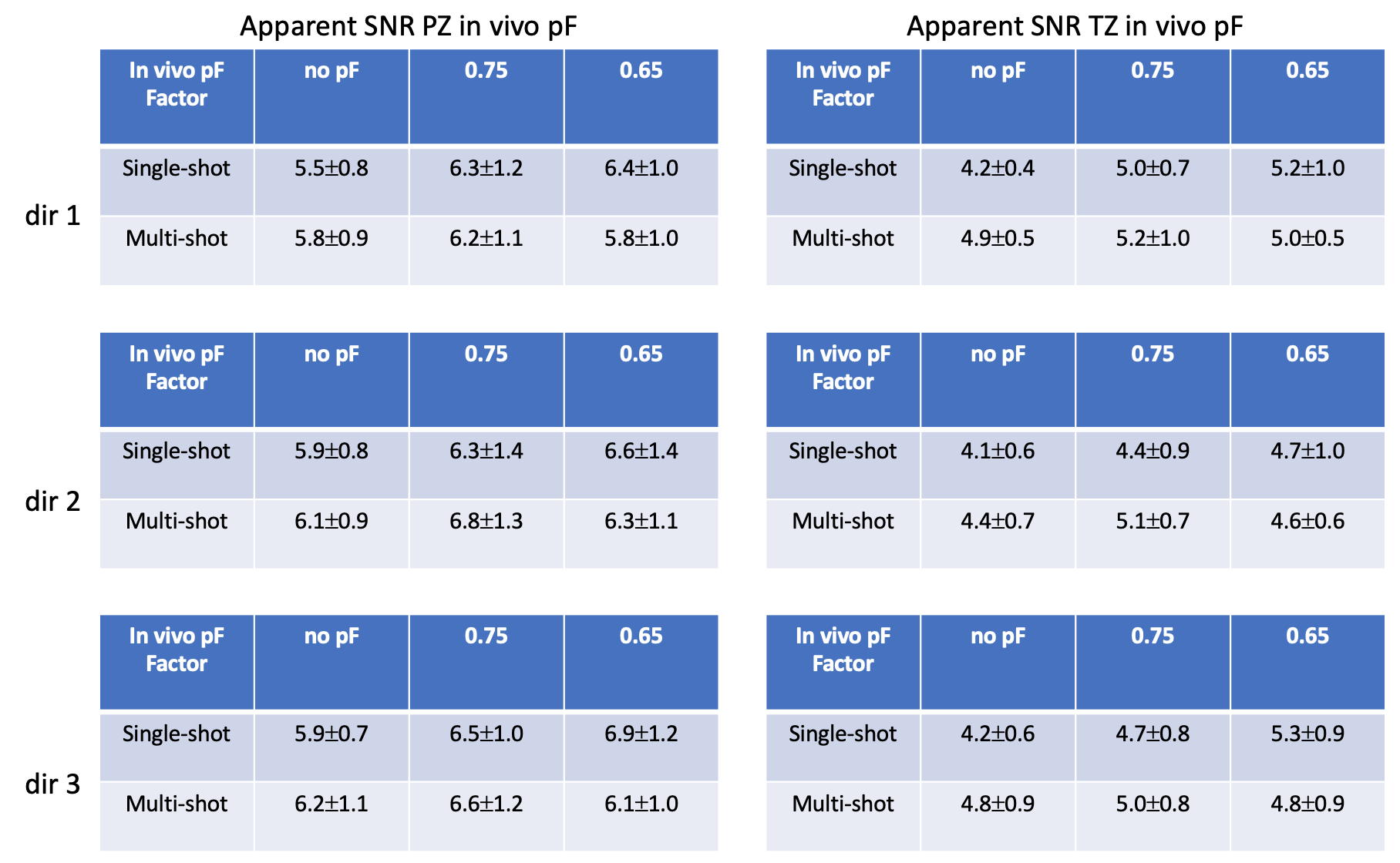

Supplement: Supplementary file 8 — (TIFF 7949 kb) [file 10334_2024_1162_MOESM8_ESM.tiff]

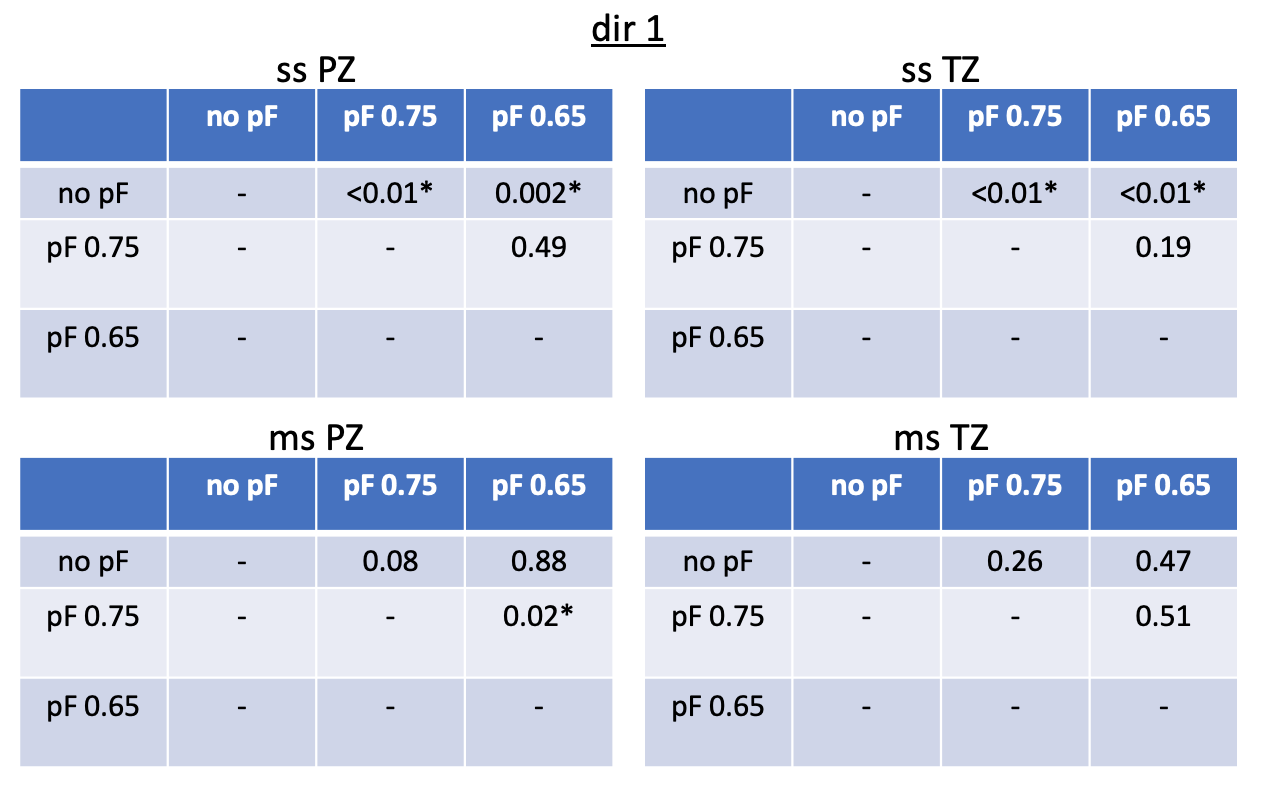

Supplement: Supplementary file 9 — (TIFF 3923 kb) [file 10334_2024_1162_MOESM9_ESM.tiff]

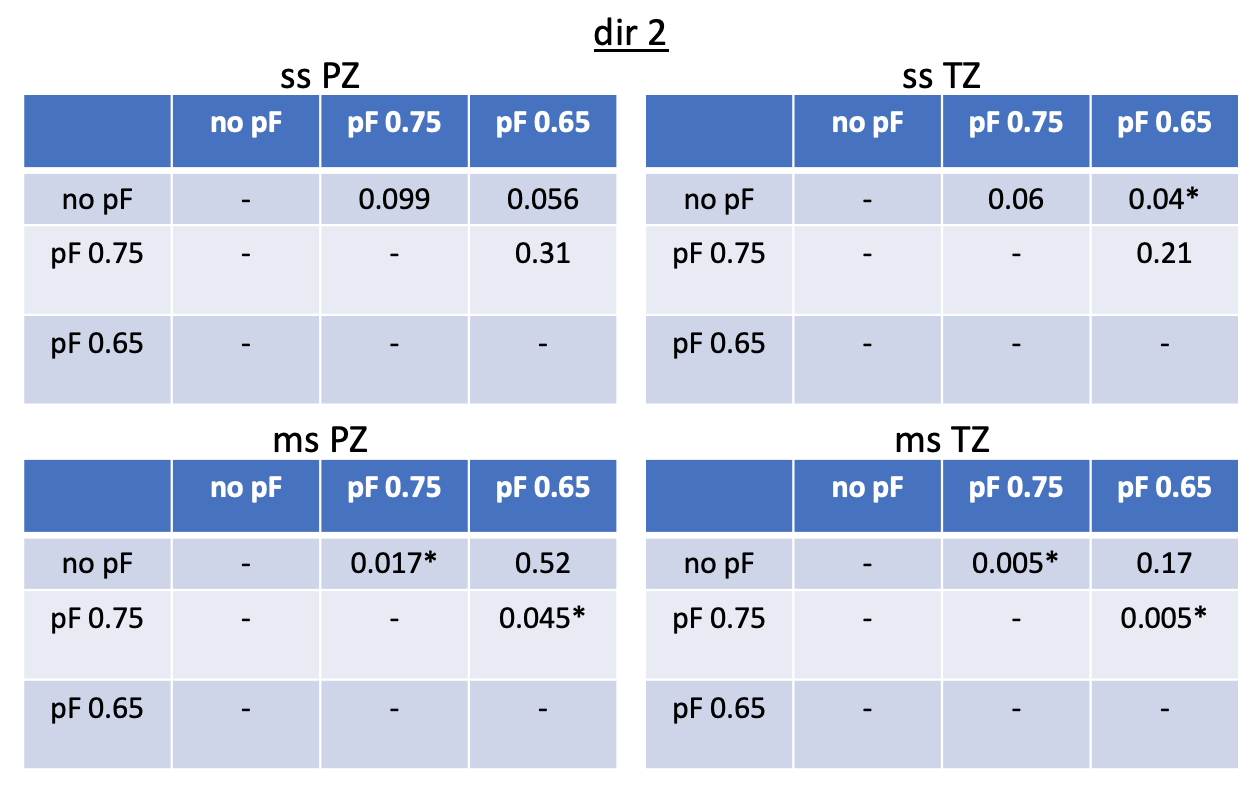

Supplement: Supplementary file 10 — (TIFF 3876 kb) [file 10334_2024_1162_MOESM10_ESM.tiff]

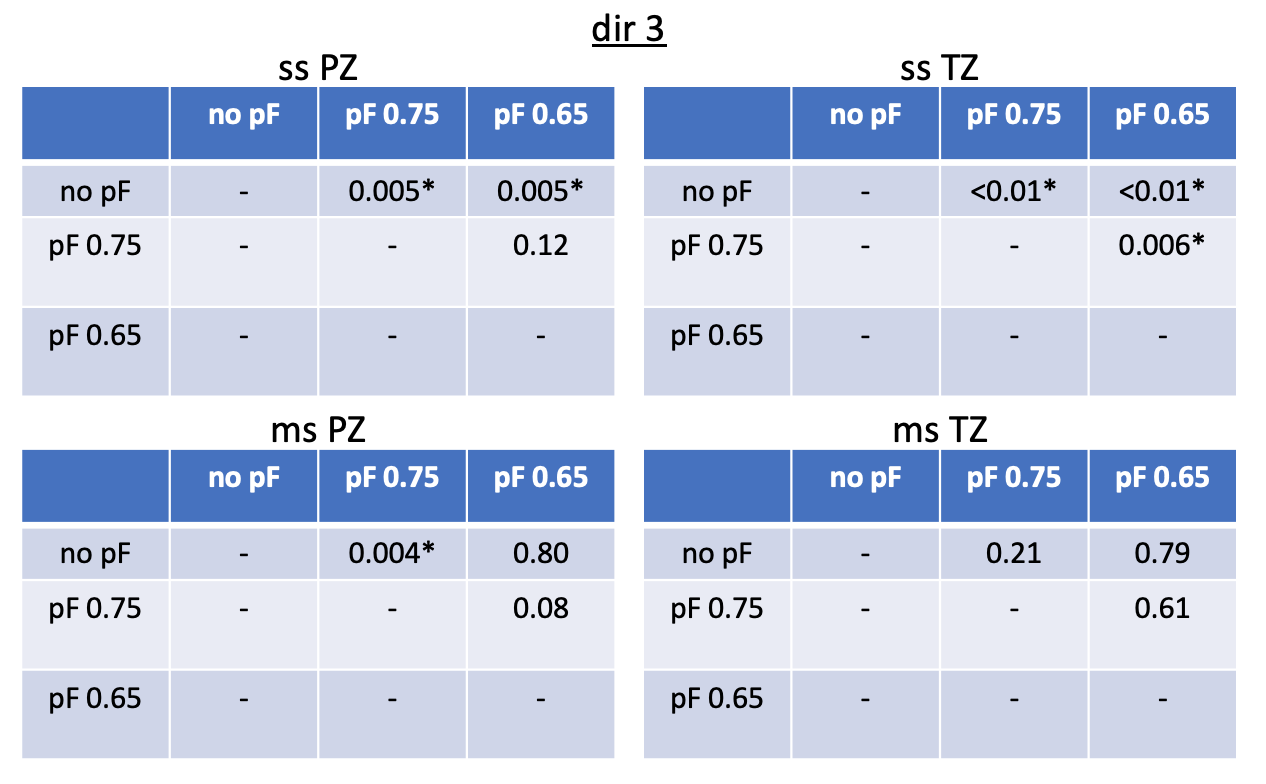

Supplement: Supplementary file 11 — (TIFF 3881 kb) [file 10334_2024_1162_MOESM11_ESM.tiff]

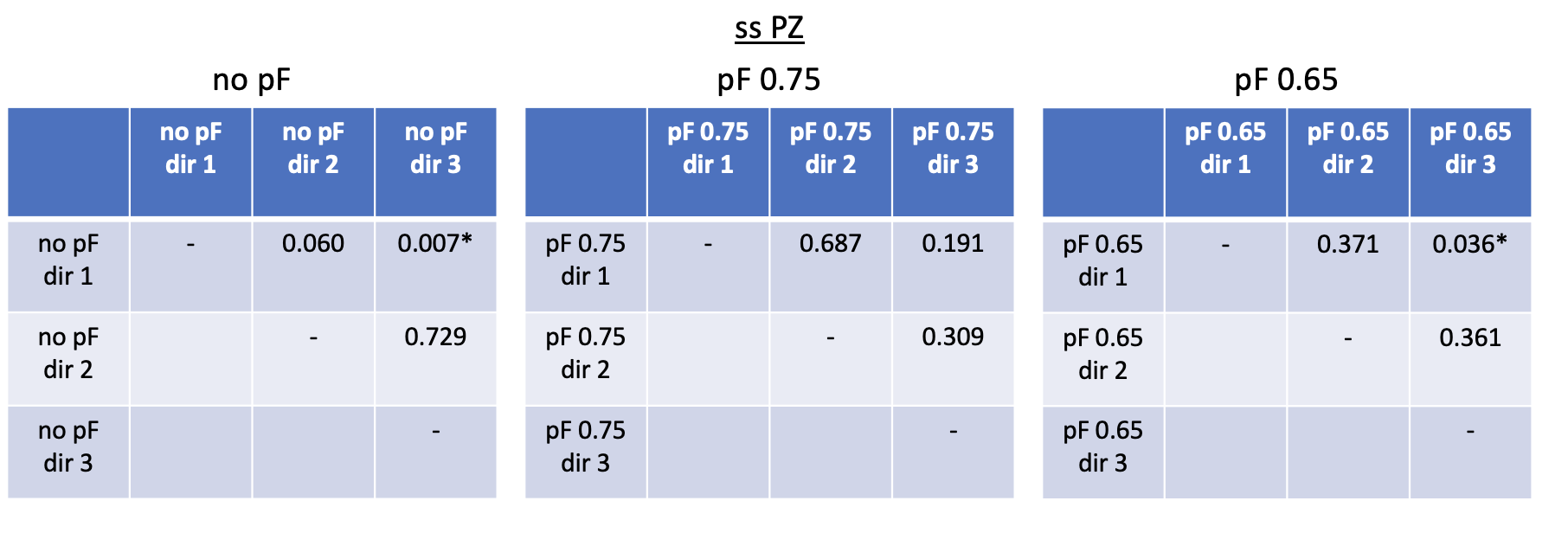

Supplement: Supplementary file 12 — (TIFF 4364 kb) [file 10334_2024_1162_MOESM12_ESM.tiff]

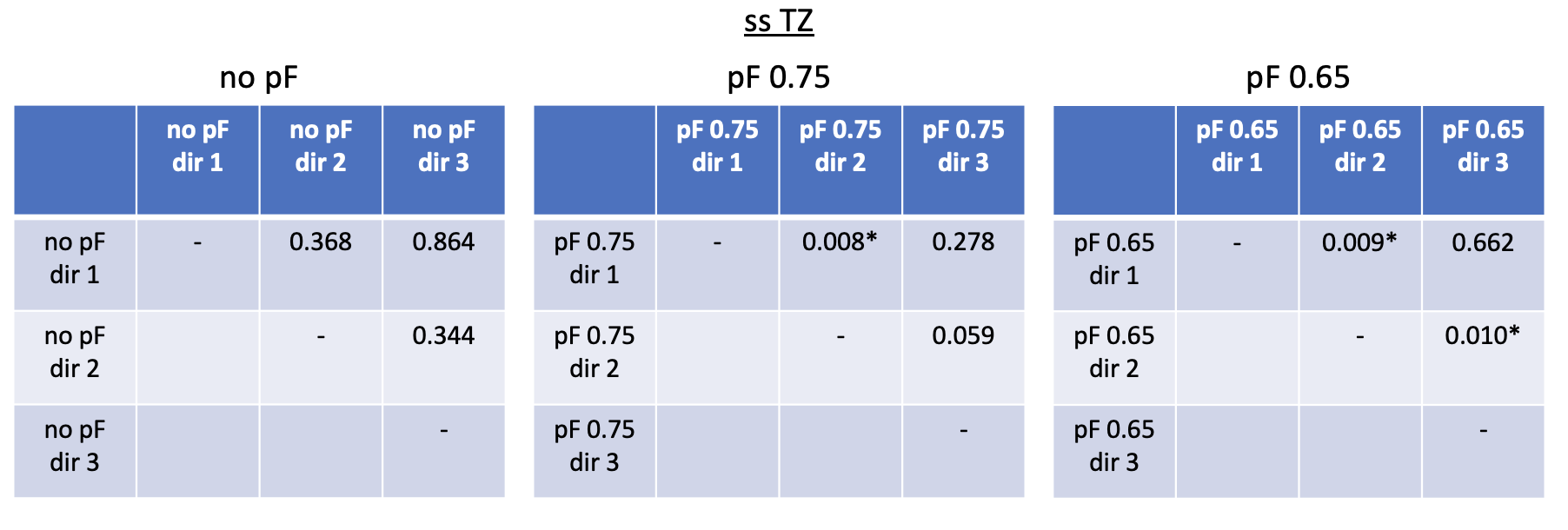

Supplement: Supplementary file 13 — (TIFF 4303 kb) [file 10334_2024_1162_MOESM13_ESM.tiff]

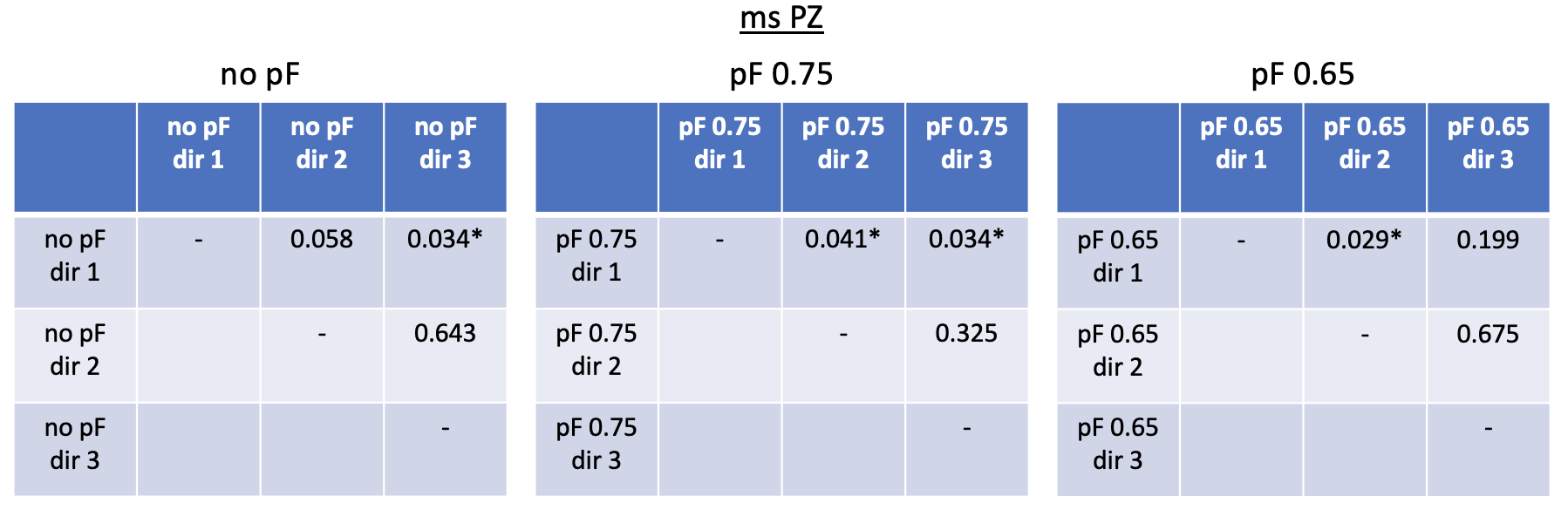

Supplement: Supplementary file 14 — (TIFF 4218 kb) [file 10334_2024_1162_MOESM14_ESM.tiff]

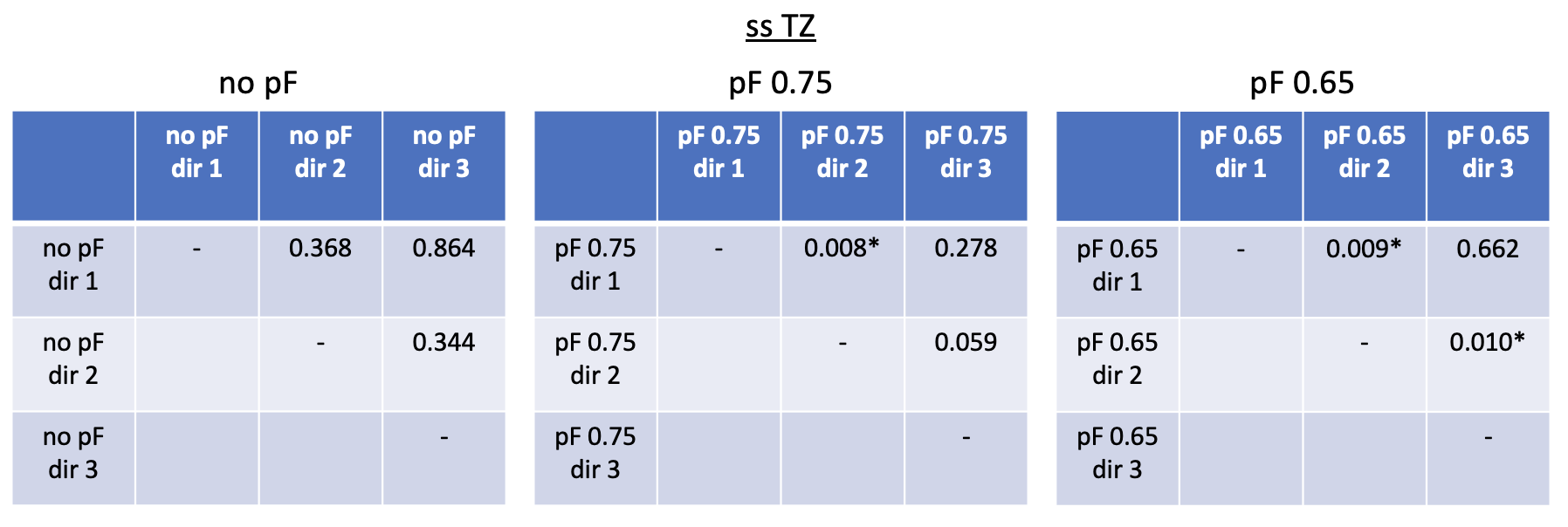

Supplement: Supplementary file 15 — (TIFF 4242 kb) [file 10334_2024_1162_MOESM15_ESM.tiff]

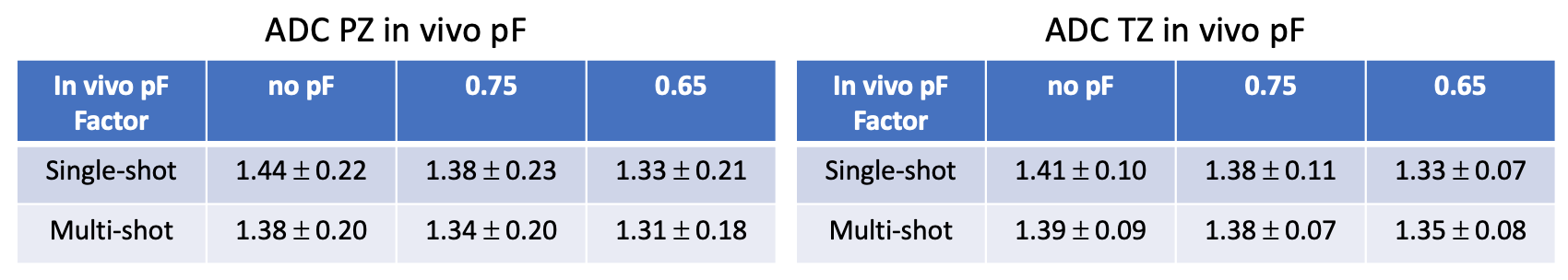

Supplement: Supplementary file 16 — (TIFF 2022 kb) [file 10334_2024_1162_MOESM16_ESM.tiff]

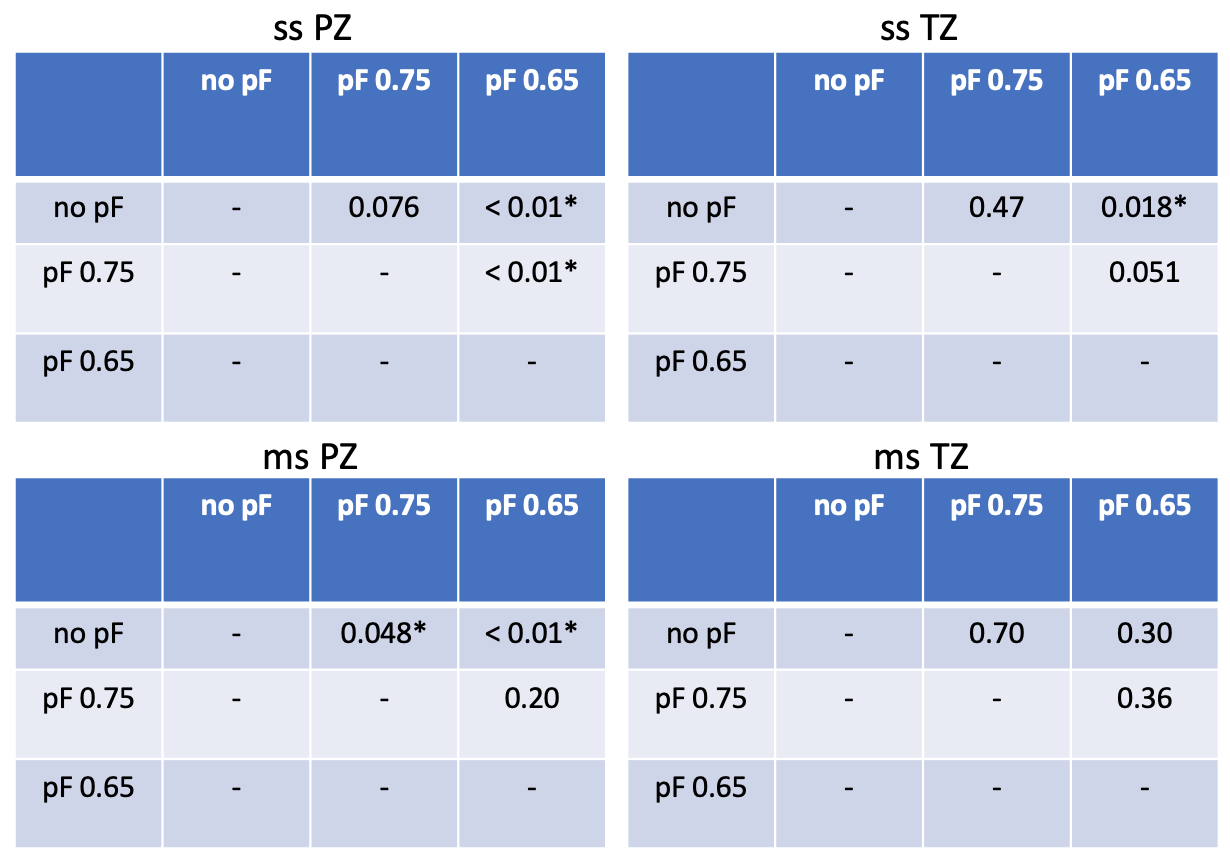

Supplement: Supplementary file 17 — (TIFF 4155 kb) [file 10334_2024_1162_MOESM17_ESM.tiff]
